# Structure–Transport Relationships of Water–Organic Solvent Co-transport in Carbon Molecular Sieve (CMS) Membranes

**DOI:** 10.1021/acs.iecr.3c02519

**Published:** 2023-10-30

**Authors:** Young Hee Yoon, Yi Ren, Akriti Sarswat, Suhyun Kim, Ryan P. Lively

**Affiliations:** School of Chemical and Biomolecular Engineering, Georgia Institute of Technology, Atlanta, Georgia 30332, United States

## Abstract

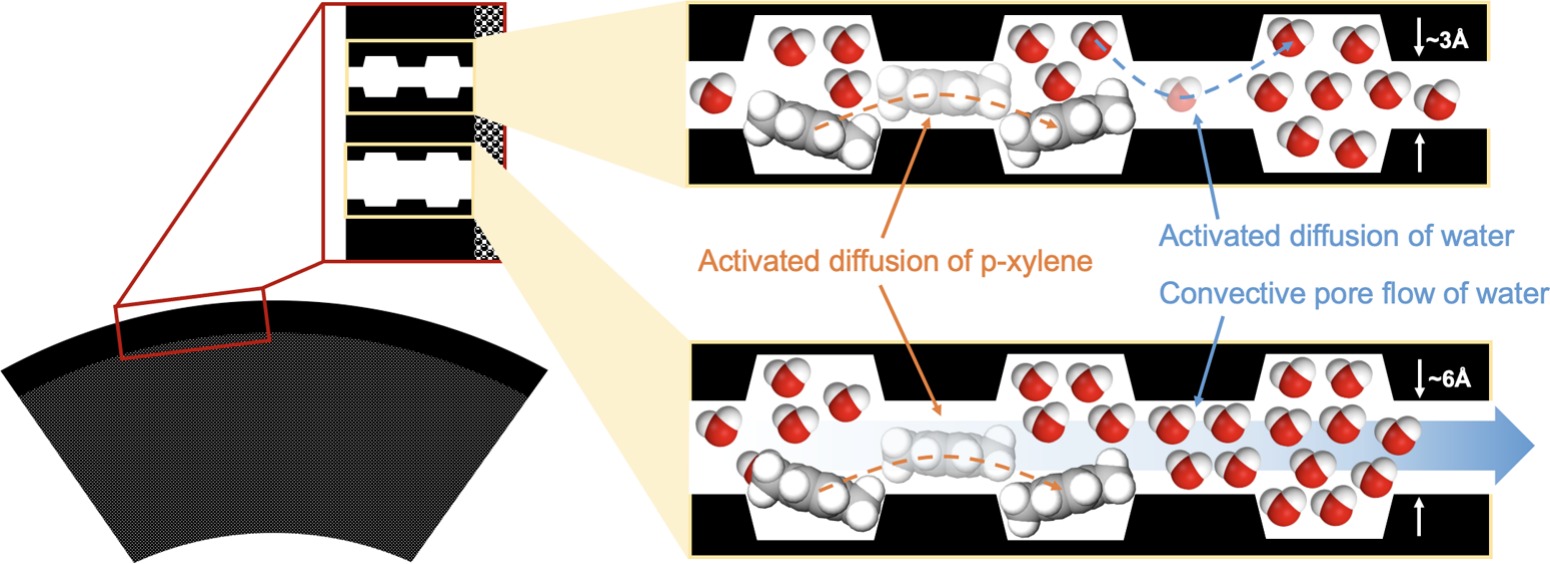

We explore the effects of the carbon molecular sieve (CMS) microstructure on the separation performance and transport mechanism of water–organic mixtures. Specifically, we utilize PIM-1 dense films and integrally skinned asymmetric hollow fiber membranes as polymer precursors for the CMS materials. The PIM-1 membranes were pyrolyzed under several different pyrolysis atmospheres (argon, carbon dioxide, and diluted hydrogen gas) and at multiple pyrolysis temperatures. Detailed gas physisorption measurements reveal that membranes pyrolyzed under 4% H_2_ and CO_2_ had broadened ultramicropore distributions (pore diameter <7 Å) compared to Ar pyrolysis, and pyrolysis under CO_2_ increased ultramicropore volume and broadened micropore distributions at increased pyrolysis temperatures. Gravimetric water and *p*-xylene sorption and diffusion measurements reveal that the PIM-1-derived CMS materials are more hydrophilic than other CMS materials that have been previously studied, which leads to sorption-diffusion estimations showing water-selective permeation. Water permeation in the vapor phase, pervaporation, and liquid-phase hydraulic permeation reveal that the isobaric permeation modes (vapor permeation and pervaporation) are reasonably well predicted by the sorption-diffusion model, whereas the hydraulic permeation mode is significantly underpredicted (>250×). Conversely, the permeation of *p*-xylene is well predicted by the sorption-diffusion model in all cases. The collection of pore size analysis, vapor sorption and diffusion, and permeation in different modalities creates a picture of a combined transport mechanism in which water—under high transmembrane pressures—permeates via a Poiseuille-style mechanism, whereas *p*-xylene solutes in the mixture permeate via sorption-diffusion.

## 1 Introduction

Global water demands are continuously growing due to economic development and population growth.^1^ Moreover, climate change exacerbates water stress and results in water quality degradation. Conventional water purification methods are insufficient to meet the increasing demand, necessitating secondary and tertiary water treatment techniques.^2^ Industrial wastewater, in particular, contains many harmful organic contaminants, including BTEX compounds (benzene, toluene, ethylbenzene, and xylenes) that dissolve in water.^3^ These compounds pose environmental and health risks,^4,5^ emphasizing the need for their safe and effective removal from wastewater. Traditional thermally driven separation methods, such as distillation, drying, and evaporation, can effectively remove organic contaminants but are energy-intensive and often unsuitable for wastewater treatment. Nonthermal methods provide alternatives that minimize the utilization of heat for a phase change. These methods include adsorbents,^4^ catalytic oxidation,^6^ air stripping,^7^ and membrane separation.^8^ Activated carbon removes soluble BTEX but requires frequent regeneration, posing economic and logistical challenges for high-concentration organics (>100 ppm).^3^ Catalytic oxidation is effective but requires careful implementation due to the use of harmful chemicals. Air stripping also efficiently removes the volatile organic solvents but needs post-treatment to prevent simply transferring the water pollution problem into an air pollution problem. Membrane-based separation is potentially highly efficient, safe, easy to operate, and cost-effective for the treatment of dissolved organics in wastewater.

**Figure 1:**
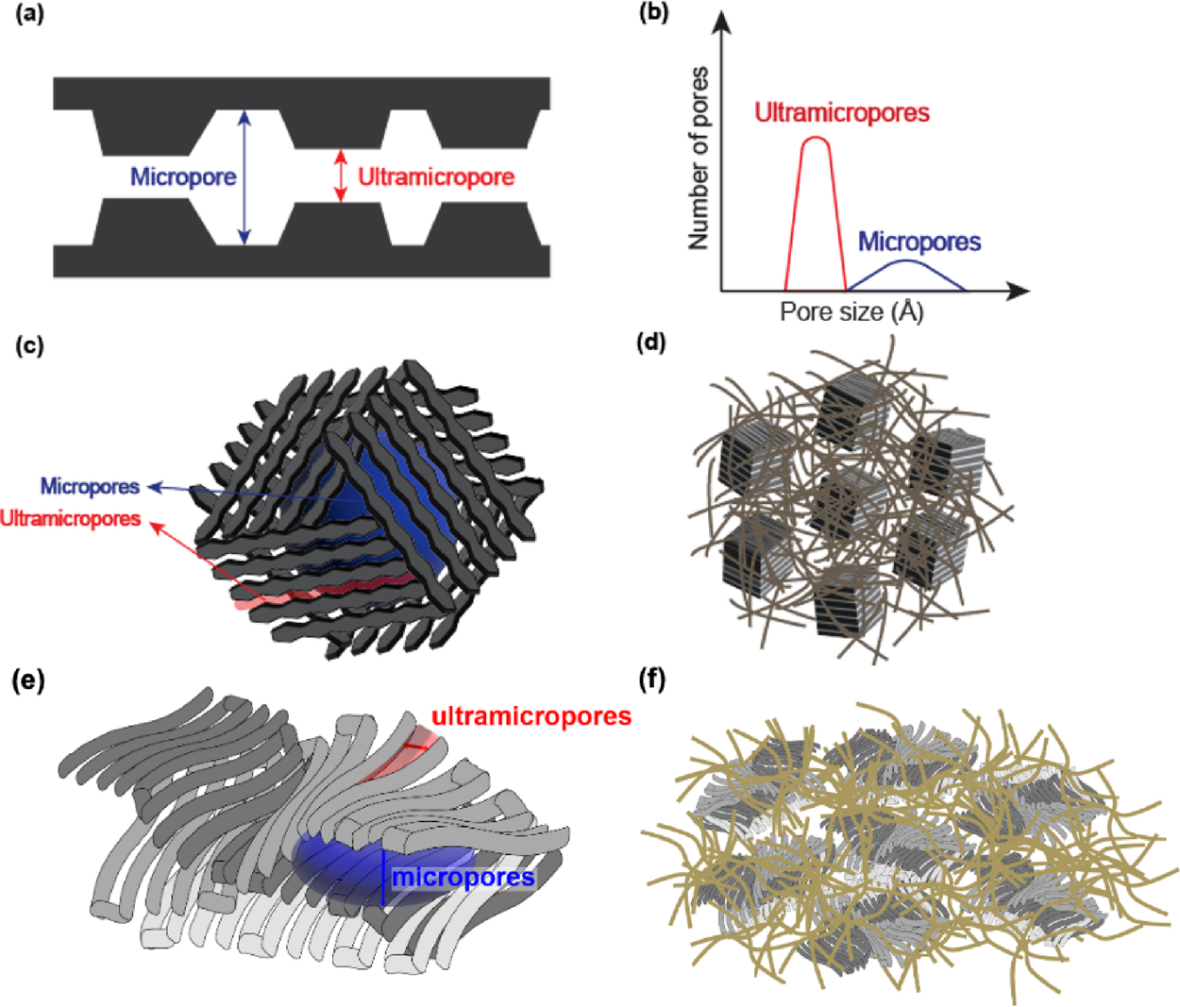
Hypothesized microstructure of CMS. (a) Classical idealized slit-like bimodal distribution of CMS micropores. (b) Generalized bimodal pore size distribution of CMS consisting of ultramicropores and micropores. (c) CMS microstructure hypothesis from Koros et al. showing the arrangement of carbonaceous plates into microporous cells.^27,31^ (d) The microporous cells are hypothesized to be surrounded by a disordered arrangement of graphenic strands (also called orphan strands) that did not assemble into plate-like structures.^28^ (e) Proposed sp^2^ carbon nanoribbon plates of PIM-1 pyrolysis products randomly intercalated and packed to form micropores. (f) Schematic of multiple units of packed/entangled graphenic nanoribbons that compose micropores and ultramicropores enclosed by the continuous phase of randomly dispersed graphenic nanoribbon strands. Panels (a)–(c) are reproduced or adapted with permission from ref (22). Panels (d)–(f) are reproduced or adapted with permission from ref (29). Copyright 2023 Elsevier.

Carbon molecular sieves (CMS) have shown great potential as high-performance membrane materials in gas and organic solvent separation due to their thermal and chemical stability as well as scalability.^9−17^ Various types of precursors including polyimides^18,19^ and cellulose-based polymers^20,21^ have been investigated for the CMS membranes for gas separations. Recent studies have also highlighted the potential of CMS membranes for treating dissolved organics in water via an organic selective separation.^22,23^ A critical advantage of CMS as a membrane material is that its pore size distribution (PSD) can be precisely engineered by altering the polymer precursors and pyrolysis conditions.^24−26^ CMS membranes offer tailored rates of guest molecule mobility within the microporous environment, offering the potential to address specific separation challenges encountered in wastewater remediation. CMS materials are amorphous and carbonaceous, with a rigid microporous structure derived from a pyrolyzed polymeric precursor. A microstructural development hypothesis for CMS materials has been proposed by Koros et al.^27,28,31^ to describe the precisely controlled bimodal pore size distribution in the amorphous carbon material. According to this hypothesis, the CMS pyrolysis typically involves three steps: (1) ramping, (2) soaking, and (3) cooling.^27,28^ During the first stage, the polymer precursor undergoes fragmentation and aromatization, thus forming rigid and aromatized “strands”. These short strands exhibit mobility at pyrolysis temperatures and imperfectly align in parallel to create imperfect “plates”. During the soaking and cooling steps, these plates further assemble into imperfect microporous “cell” structures. The neighboring cells then merge, forming a repeating pattern of micropore cells with ultramicroporous slit walls (Figure 1c,d). Moreover, a recent study on CMS derived from polyimide has introduced a new structural feature called “orphan strands”,^28^ which form a minority continuous network of disordered ultramicropores located between the microporous cells (Figure 1d). Another recent hypothesis on the PIM-1-derived CMS structure^29^ proposes a similar hypothetical structure that comprises sp^2^-hybridized carbon nanoribbons oriented to form two-dimensional curved carbon sheet layers (Figure 1e,f). The imperfect packing and weaving between these curved layers create micropores, and the slits between the nanoribbons, which compose the carbonaceous plane, become ultramicropores.

The CMS pore structures can be visualized as slit-like pores with interconnected bimodal PSD (Figure 1a), offering both high selectivities from the ultramicropores and high permeability from larger micropores. The processability of the polymer precursor allows CMS membranes to be manufactured in commercially relevant form factors, including hollow fiber membranes. This study utilizes a polymer of intrinsic microporosity-1 (PIM-1) as the precursor due to its known successful fabrication into hollow fiber membranes for liquid separation^13,23,30^ and its versatility in adjusting the microstructure through adjustments in the pyrolysis atmosphere.^13^

Previous work^22^ investigated the transport of water and *p*-xylene vapors in polyvinylidene fluoride (PVDF)-derived CMS membranes using a sorption-diffusion (SD) transport model. Sorption and diffusion coefficients were experimentally measured via gravimetric vapor uptake measurements, and permeabilities were calculated using the SD model with the appropriate driving forces. Experimental permeabilities were then measured in a Wicke–Kallenbach (WK) vapor permeation system and were compared to the calculated values, which suggested that water and *p*-xylene transport in PVDF-derived CMS follows the SD mechanism. Despite the molecular size difference between water (2.6 Å) and *p*-xylene (5.8 Å), the PVDF-CMS membranes exhibited selectivity toward *p*-xylene and behaved as organic concentrating membranes in water–organic separation. This type of behavior was also recently observed in the liquid phase for the separation of water and dimethylformamide.^23^

This study investigates the structure–transport relationship of water–*p*-xylene transport in CMS materials. PIM-1-derived CMS membranes were fabricated with a tunable PSD through pyrolysis conditions. The sorption and diffusion coefficients of water were measured via gravimetric vapor uptake experiments in an exemplar PIM-1-derived CMS membrane, and the SD permeabilities were calculated and compared with the experimental vapor permeabilities. Vapor permeation experiments were conducted for water and *p*-xylene in various CMS membranes, providing insights into their separation capabilities in a variety of membranes with different PSD and surface chemistries. Additionally, we explore the transport mechanism of water and *p*-xylene in a PIM-1-derived CMS membrane in reverse osmosis (RO), pervaporation (PV), and vapor permeation (VP) modalities.

## 2 Theory and Background

### 2.1 Molecular Transport across Microporous CMS Membranes

Permeability () and permselectivity (α) are the two main parameters used to describe the ideal performance of a sorption-diffusion membrane. Permeability represents the intrinsic productivity of the membrane material, while permselectivity is indicative of the separation efficiency. The single-component permeability of component *i* () can be determined by measuring the flux of *i* (*N_i_*) and normalizing with the membrane selective layer thickness () and the transmembrane fugacity across the membrane (Δ*f_i_*), which is the appropriate driving force for molecular transport across microporous membranes with ultramicropores (<7 Å) present (eq 1). The fugacity is simply a mathematical manipulation of the chemical potential, and we favor its use in membrane calculations, as the fugacity conveniently goes to zero when the system pressure goes to zero (unlike the chemical potential). The ideal permselectivity of permeants *i* and *j* (α_*ij*_) is defined as the ratio of the permeabilities when the membrane downstream is in a vacuum (eq 2). For gas and vapor separation, the transmembrane fugacity can often be approximated as the difference in the partial pressure across the membrane.
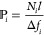
1
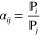
2

Microporous CMS membranes commonly transport guest molecules through a sorption-diffusion mechanism. In the SD model, permeability can be expressed as a product of a kinetic factor, a diffusion coefficient (*D*_*i*_), and a thermodynamic factor, a sorption coefficient () (eq 3). The model posits that penetrants are adsorbed in microporous sites in the CMS. Then, the penetrants diffuse through the membrane by making size-dependent jumps through the ultramicropore windows. The ultramicroporous windows play a key role by acting as kinetic restrictions in the diffusion process, thus enabling an effective distinction between penetrants of similar-size. The diffusive transport is driven by the chemical potential gradient (or the fugacity gradient),^32^ which is well represented by the guest concentration gradient in CMS membranes.^33−35^ The ideal permselectivity in the SD model can be expressed as the ratio of pure permeabilities or the product of diffusion selectivity and sorption selectivity (eq 4).
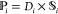
3
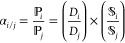
4

In this study, the transport of water and *p*-xylene in various PIM-1-derived CMS was studied by the SD transport model. The diffusion and sorption behavior of water and *p*-xylene in CMS was experimentally studied in vapor gravimetric sorption experiments to obtain *D* and . The detailed derivation for the mathematical expressions of *D* and  is provided in the Supporting Information (S 1). The permeability of water and *p*-xylene was then experimentally measured in the WK vapor permeation system to test whether the SD model governs the transport in the PIM-1-derived CMS. Mixture permeation experiments were also conducted to observe mixture transport behaviors.

A more simplified equation was employed to estimate the fluxes of more complex membrane separation modalities, such as RO and PV, in which the driving forces and boundary conditions are more complicated than those found in vapor permeation. The SD flux equation for a diffusive molar flux (*J*_*i*_) (eq 6) was derived^23^ from a simplified MS expression (eq 5).
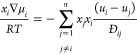
5
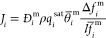
6where *x*_*i*_ is the mole fraction of component *i* in the membrane, ∇μ_*i*_ is the chemical potential gradient of *i* across the membrane, *u*_*i*_ is the velocity of *i* in the membrane, *Đ*_*ij*_ is the mutual MS diffusion coefficient between components *i* and *j*, where *i* or *j* could be the adsorbates or the membrane, and  is the MS diffusivity of *i* in the membrane, which is also a thermodynamically corrected diffusivity (see eq 8). Here, ρ is the density of the membrane,  is the saturation loading of *i* in the membrane,  is the average fractional occupancy of *i* in the membrane,  is the transmembrane fugacity of *i* in the membrane,  is the thickness of the membrane selective layer, and  is the average fugacity of *i* in the membrane. The detailed assumptions used in the simplification are also provided in the Supporting Information (S 1). The separation performance in the RO system was expressed in terms of the separation factor (β_*ij*_) (eq 7).
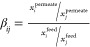
7where  and  are mole fractions of *i* in permeate and feed, respectively.

## 3 Methods

### 3.1 Fabrication of PIM-1-Derived CMS Membranes

PIM-1 was synthesized using the low-temperature polycondensation technique developed by Budd et al.^36^ A more detailed explanation for the PIM-1 synthesis is provided in the Supporting Information (S2. 2). Dense film PIM-1-derived CMS membranes were fabricated and applied in the VP system, and the asymmetric PIM-1-derived CMS hollow fiber membranes were fabricated using dry-wet spinning^30^ and applied for RO and PV membrane systems. Detailed polymer membrane fabrication methods are provided in the Supporting Information (S2. 3). The CMS samples were pyrolyzed in various gas atmospheres and at different maximum pyrolysis temperatures. The polymer membranes were pyrolyzed in a three-zone furnace (MTI Corporation) using a quartz tube sealed on both ends (Figure S 1). Ultrahigh purity argon, 4% hydrogen balanced with argon, or bone-dry carbon dioxide was fed into the tube at 200, 200, and 380 sccm flow rates, respectively, and were purged overnight. An oxygen analyzer was installed downstream of the quartz tube, and the oxygen level was monitored to stay below 2 ppm before starting the pyrolysis protocol. The details of the pyrolysis temperature ramping profile for different precursors and different final temperatures are provided in Table S 1. Between every pyrolysis, the quartz tube was rinsed with acetone and baked off in air at 800 °C for >2 h to remove impurities from the previous experiments. The fabricated CMS samples are named in this work as **[precursor]_[pyrolysis atmosphere]_[final temperature in °C]_CMS**.

### 3.2 Material Characterization

X-ray photoelectron spectroscopy (XPS) was conducted on CMS powder samples, which are ground from dense CMS films, to obtain the average chemical composition of the membrane materials. Scanning electron microscopy (SEM) micrographs were taken on the membrane cross-sections to measure the thickness of the dense filmsm and the diameter of the hollow fiber membranes. Carbon dioxide physisorption at 273 K was performed on the CMS materials to obtain the microporous volume of the carbon materials and the microporous pore size distribution in the <10 Å range. A detailed characterization description is provided in the Supporting Information (S2. 4).

### 3.3 Vapor Sorption Measurements

Vapor sorption isotherms of pure water and *p*-xylene on PIM-1-derived and PVDF-derived CMS were measured by using an automated gravimetric vapor sorption instrument, VTI-SA+ (TA Instruments, New Castle, DE). The vapor uptakes from 5 to 75% relative pressures were obtained at 35 °C, and the water uptakes at unit activity were obtained by soaking the dense CMS film membranes in liquid water at 35 °C. The unit activity water uptake was used to estimate the *p*-xylene uptakes at unit activity, assuming that the pores are fully occupied by the liquid adsorbates and that the adsorbates will exhibit liquid-like densities within the micropores. A detailed explanation on the gravimetric vapor sorption experiment is provided in the Supporting Information (S2. 5)

The vapor sorption measurements provided both the transient mass uptake data and the isotherms, which were used to obtain the diffusion and sorption coefficients (eq (S2) and (S7)). The transient mass uptake data were normalized and then fit to a Fickian mass transfer equation to obtain the transport diffusion coefficient, *D*. The Maxwell–Stefan (MS) diffusivities, *Đ*, are ideally independent of guest loading, and they were obtained by thermodynamically correcting (Γ) the transport diffusivities, *D*, which are guest loading-dependent (eq 8). However, in real scenarios, the MS diffusivities can still depend on the loading in strong-confinement or hybrid-confinement scenarios.^33,37^ Therefore, the MS diffusivities used in this work are obtained at the corresponding average loading estimated based on the experimental boundary conditions of the membrane upstream and downstream fugacities.
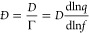
8

### 3.4 Permeation Experiment

#### 3.4.1 Wicke–Kallenbach (WK) Vapor Permeation Experiment

**Figure 2:**
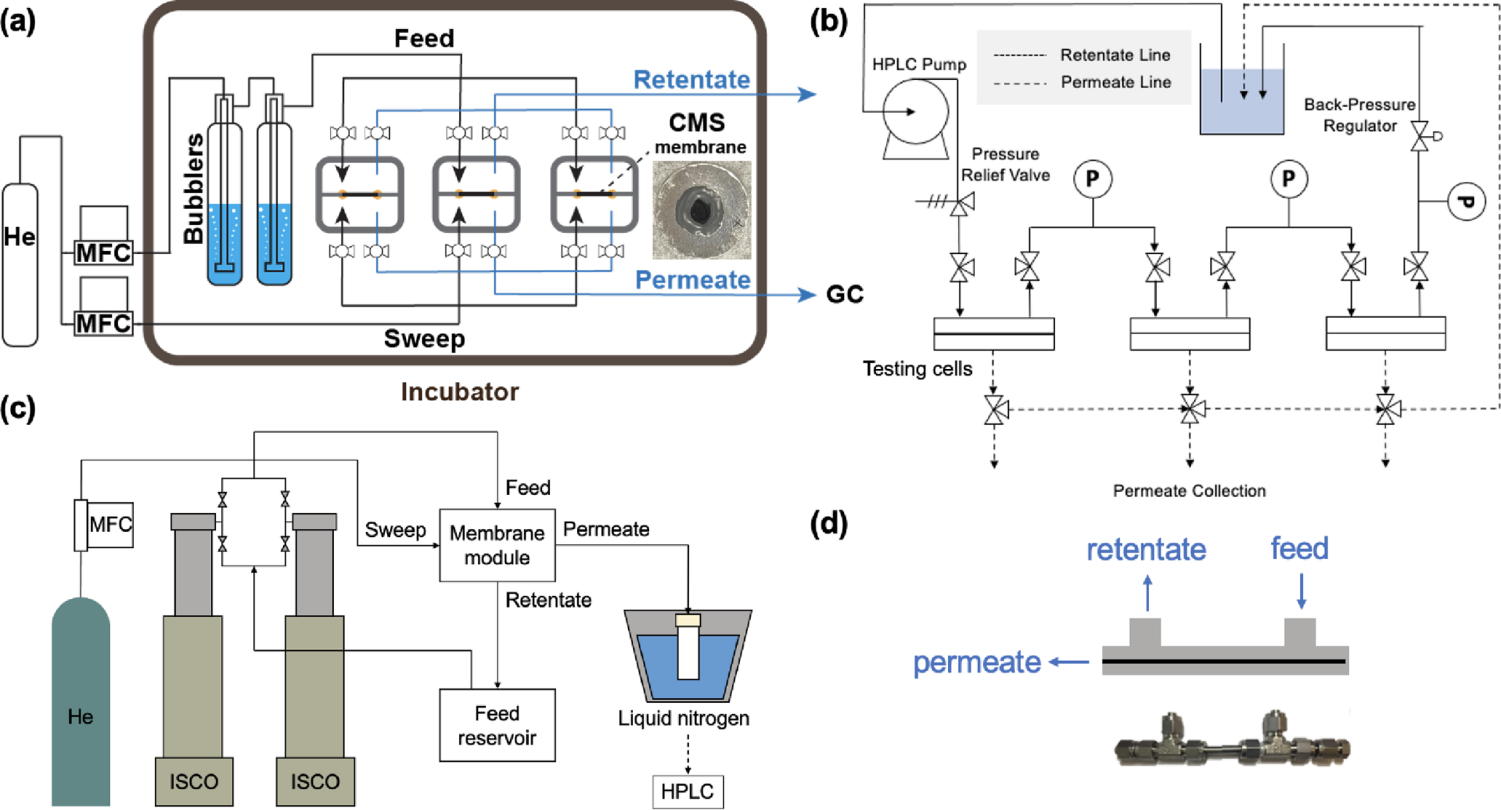
Simplified schematics of permeation experiments. (a) Schematic of Wicke–Kallenbach (WK) vapor permeation experiment. (b) Schematic of crossflow liquid permeation systems driven by an HPLC pump. (c) Schematic of the crossflow pervaporation system driven by a dual syringe pump crossflow system. (d) Hollow fiber membrane modules used in the liquid permeation systems. The feed is fed to the shell side, and the permeate is collected on the bore side of the hollow fiber membranes.

The pure component and mixture permeation experiments for the water and *p*-xylene vapors were performed in a Wicke–Kallenbach (WK) permeation device (Figure 2a). The WK method is suitable for studying fundamental transport mechanisms. WK provides a simple and straightforward experimental measurement while avoiding many nonidealities observed in real separation systems. WK is an isobaric and isothermal system, where the permeation is driven by the concentration gradient across the upstream and downstream of the membrane. Helium gas was fed through a bubbler containing pure water, pure *p*-xylene, or a water/*p*-xylene mixture. Two bubblers were connected in series to ensure the generation of a saturated vapor stream, and glass wool demisters were installed in each saturator outlet to avoid droplet deposition on the membrane surface. The saturated vapor stream from the bubblers was fed into the membrane. The CMS membranes were masked using aluminum tape, supported with filter paper, and sealed with an epoxy (J-B Weld 8272 MarineWeld) to install CMS membranes in the membrane cells and seal with o-rings without breaking the membranes. The downstream was fed with helium sweep gas to create a near-zero activity environment and induce the maximum activity (or fugacity) gradient across the membrane. The upstream helium flow rate was kept at 20 sccm, and the downstream helium flow rate was kept at 5 sccm. The permeability was calculated using eq 9, where *ṅ*_A_ is the molar flow rate,  is the thickness of the CMS membrane, *A* is the effective area of the CMS membrane, and  and  are the partial pressure of water or *p*-xylene upstream and downstream. The molar flow rate was measured using a bubble flow meter installed on the permeate stream. The thickness of the CMS membrane was measured by SEM, and the effective permeation area was measured using ImageJ software. The partial pressures,  and , were obtained by measuring the stream composition using gas chromatography (GC, Agilent, Santa Clara, CA). More than two membrane samples were installed in the system in parallel and tested for reproducibility.
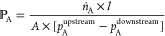
9

#### 3.4.2 Liquid Permeation Experiments in Reverse Osmosis (RO) and Pervaporation (PV) Systems

Asymmetric hollow fiber CMS membranes were tested using a custom-made bench-scale crossflow permeation system for reverse osmosis separation and a custom-made bench-scale pervaporation permeation. The crossflow permeation system was pressurized by a high-pressure HPLC pump (Azura P 4.1S, Knauer) (Figure 2b). The liquid permeation experiments were conducted at room temperature. Deionized (DI) water saturated with *p*-xylene (∼290 ppm) liquid was used as a feed mixture. Prior to running high-pressure liquid permeation experiments on the membranes, the CMS membranes were wetted with the feed solution on the shell and the bore side to condition the module overnight. The feed mixture was fed to the shell side of the membranes (Figure 2d), and the retentate was cycled back to the feed reservoir to maintain the feed mixture concentration. Once the membrane modules were installed on the crossflow systems, the feed solution was circulated for 24 h to remove air bubbles in the systems at ambient pressure before exposure to high pressure. The membranes were slowly pressurized to minimize the stress on the membranes.

The first sample collection (∼0.5 mL ≫ 10 times the downstream volume) was discarded to purge out the bore side solution, which was injected prior to the permeation experiment, and to ensure the sample collection was at a steady state. The mass of an empty vial before permeation, the mass of the vial with permeate after the permeation, and the time for permeation were recorded to obtain the permeate flow rate. The effective membrane area was obtained by measuring the outer diameter of the hollow fiber membranes in SEM (SU 8010) and by measuring the effective fiber length. For mixture permeation, the feed was sampled at the start and the end of the permeate collection. The compositions of the feed mixture were measured, and the average was used as the feed composition for the permeate sample. The feed and permeate compositions were measured by high-performance liquid chromatography (HPLC, Agilent).

The pure water pervaporation experiments were conducted in a custom-built crossflow pervaporation system (Figure 2c). Two high-pressure mechanical syringe pumps (500D, Teledyne Isco) were operated in a dual-pump mode for a continuous flow system. The hollow fiber CMS membranes were tested in the reverse osmosis modality before being tested in pervaporation. After the RO experiments, the wet fiber membranes were stored in water to prevent damage to the fiber membranes during drying. Then, similar to the reverse osmosis setup, the DI water feed was fed to the shell side of the membrane at a flow rate of 10 mL/min. The downstream of the membrane was purged with a sweep He gas at a flow rate of ∼18 sccm to create a chemical potential gradient across the membrane. The pervaporation experiments were also conducted at room temperature. The initial permeate collection (∼0.5 mL ≫ 10 times the downstream volume) was discarded to purge out the bore side solution, which was injected prior to the experiment, and to collect the permeate at a steady state. The permeate was collected in four 60 mL septum vials connected in series to ensure the collection of the condensed permeate. The collection vials were contained in a liquid nitrogen cooling bath to condense the vapor permeates. The mass of an empty vial before permeation, the mass of the vial with condensed permeate after the permeation, and the time for permeation were recorded to obtain the permeate flow rate.

## 4 Results and Discussion

### 4.1 Material Characterization

The ultramicropores and micropores below 10 Å of various CMS samples were examined by carbon dioxide physisorption at 273 K up to 1 bar (*p*/*p*^sat^ = 0.03). The CMS microstructure was tailored by adjusting the (1) polymer precursor, (2) pyrolysis atmosphere, (3) final pyrolysis temperature (*T*_p_), and (4) pyrolysis temperature profile. First, the polymer precursor was varied between PIM-1 and PVDF. Second, different pyrolysis atmospheres were utilized, including Ar, 4% H_**2**_ balanced with Ar, and CO_**2**_. Third, the final pyrolysis temperatures of 500 and 800 °C were tested under the same pyrolysis atmosphere of CO_2_. Last, the pyrolysis temperature profile was varied by eliminating the 2 h hold step in some of the experiments. These adjustments enabled an exploration of the CMS microstructure and its dependence on the parameters of interest noted earlier. Moreover, the final pyrolysis temperatures were set to be above 500 °C based on the thermogravimetric analysis (TGA) of PIM-1 under inert gas N_2_,^38^ which showed a significant mass loss at ∼500 °C, indicating carbonization at that temperature.

**Figure 3:**
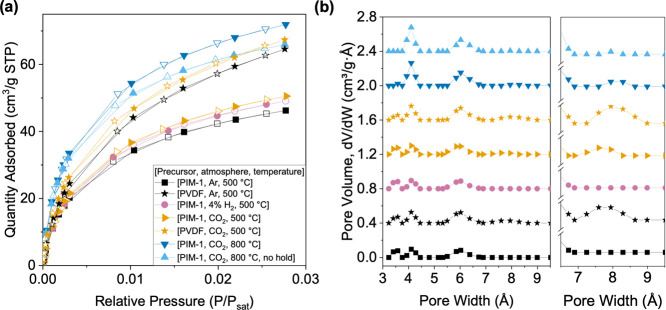
(a) Carbon dioxide isotherms on various CMS materials measured at 273 K. Solid symbols are adsorption points, and hollow symbols are desorption points. (b) Pore size distributions of the various CMS samples estimated using 2D-NLDFT. **PVDF_Ar_500_CMS** data are adapted from the previous work.^22^

Figure 3a and Table S 2 show the CO_2_ uptake (kinetic diameter = 3.3 Å) in the microporous volumes within the CMS samples at 273 K. **PIM-1_Ar_500_CMS** shows the lowest microporous volume, followed by **PIM-1_4% H_2__500_CMS**, **PIM-1_CO_2__500_CMS**, **PVDF_Ar_500_CMS**, **PIM-1_CO_2__800_no hold_CMS**, **PVDF_CO_2__500_CMS**, and **PIM-1_CO_2__800_CMS**. First, the PIM-1-derived CMSs that are pyrolyzed under different conditions were compared. By introducing 4% H_2_ in the pyrolysis atmosphere, **PIM-1_4% H_2__500_CMS** shows increased micropore volume, as observed in a previous study.^13^ PIM-1-derived CMS pyrolyzed under CO_2_ at the same pyrolysis temperature of 500 °C shows a similar micropore volume as 4% H_2_ pyrolysis; both are an evident increase relative to Ar pyrolysis at the same pyrolysis temperature. The PIM-1-derived CMS pyrolysis under CO_2_ was also conducted at an elevated temperature of 800 °C, which led to a ∼40% increase in the micropore volume at *p*/*p*^sat^ = 0.03 compared to CMS pyrolyzed under CO_2_ at *T*_p_ = 500 °C. This is an interesting observation, as the increase in pyrolysis temperature under an inert Ar environment on polyimide or PIM-1 precursors has shown more tightly packed CMS structures (i.e., narrower distributions of both micropores and ultramicropores^13,39^). Moreover, PIM-1-derived CMS pyrolysis under CO_2_ at 800 °C was conducted with no hold (**PIM-1_CO_2__no hold_800_CMS**) to understand the effect of the hold step in pyrolysis. It showed a decreased micropore volume compared to **PIM-1_CO_2__800_CMS**.

A pore size distribution analysis was conducted using the CO_2_ isotherms at 273 K and HS-2D-NLDFT (nonlocal density functional theory for heterogeneous surface) calculation (Figure 3b and Table S 3). Overall, the gas physisorption experiment suggests that the ultramicropore (in 3–4 and 5–7 Å ranges) and micropore (>7 Å) size distributions of CMS are tailorable by varying the precursor and the pyrolysis condition. Compared to the conventional Ar pyrolysis, the pyrolysis atmosphere of 4% H_2_ induces increased ultramicroporous volumes and sizes, and CO_2_ also causes an increase in the ultramicropore volume and size. An increase in the pyrolysis temperature under a CO_2_ environment also resulted in further increases in the ultramicropore and micropore volume. An interesting observation is the difference in the existence of micropores larger than 7 Å when comparing the CMS samples pyrolyzed with and without a thermal soaking step. PIM-1-CMS _CO_2__800_CMS showed the highest uptake in the micropore region (>7 Å) among the PIM-1-derived CMS. On the other hand, PIM-1_CO_2__no hold_800_CMS exhibited negligible micropores in the 7–10 Å range. Such observations align with the hypothetical structural development where the graphenic carbon strands/nanoribbons align in platelet-like orientations during thermal soaking, which then imperfectly pack to form micropores. The elimination of the hold step in pyrolysis likely resulted in a less ordered arrangement that prevents the formation of micropores within the CMS. Moreover, the PVDF-derived CMS shows a higher microporous volume compared to the PIM-1-derived CMS.

**Figure 4:**
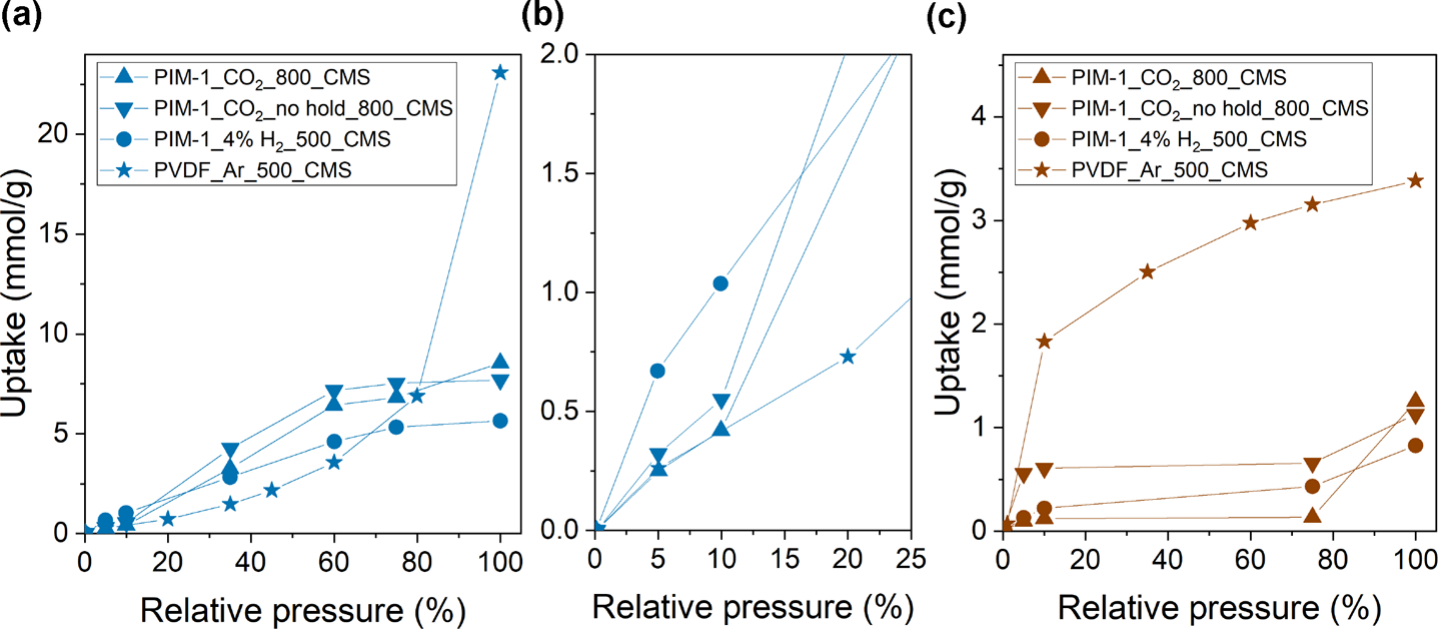
Water (blue) and *p*-xylene (brown) vapor isotherms in the range of (a, c) 0 to 1 activity and (b) 0 to 0.25 activity on PIM-1-derived CMS dense films using gravimetric vapor sorption at 35 °C. The unit activity uptakes were obtained from a liquid water-soaking experiment. The isotherms for **PVDF_Ar_500_CMS** were adapted from ref (22).

**PIM-1_4% H**_**2**_**_500_CMS**, **PIM-1_CO**_**2**_**_800_CMS**, and **PIM-1_CO**_**2**_**_no hold_800_CMS** dense film materials, which have various pore size distribution and surface chemistries, were interrogated with pure water and *p*-xylene vapor isotherms obtained at 35 °C (Figure 4). The isotherms were also compared to the water and *p*-xylene isotherms for **PVDF_Ar_500_CMS**.^22^ A distinct difference in the water isotherms was observed between PVDF-derived CMS and PIM-1-derived CMS. **PVDF_Ar_500_CMS** exhibits a distinct type 3 isotherm according to Brunauer–Deming–Deming–Teller (BDDT) classification.^40,41^ Type 3 isotherms are commonly observed for water adsorbing onto non-hydrophilic materials. The low uptake at low relative humidity shows the weak interaction between the water adsorbates and the non-hydrophilic CMS surface. As the activity increases, the uptake of adsorbates increases due to increasing adsorbate–adsorbate interaction with the already-adsorbed water molecules and forming water clusters in the sorbent material.^42^ On the other hand, the PIM-1-derived CMS materials do not exhibit such a concave-shaped isotherm, suggesting stronger interactions between the water adsorbates and these CMS materials. We speculate that the non-hydrophilic property of the PVDF-derived CMS is due to the remaining fluorine from the PVDF precursor, as observed from the XPS scans (Figure S 3).

The *p*-xylene vapor isotherms on both CMS materials show similar Langmuir-type isotherms, commonly observed for organic components adsorbing to microporous material. A distinct difference is observed where the PIM-1-derived CMS shows significantly lower *p*-xylene uptake than **PVDF_Ar_500_CMS**. Such a difference can be attributed to less pore volume available for the liquid adsorbents in these PIM-1-derived CMS samples.

### 4.2 Sorption-Diffusion Transport Study of Water on the PIM-1-Derived CMS Membrane

The transport of water and *p*-xylene in **PVDF_Ar_500_CMS** has been investigated in previous work,^18^ and it was shown to approximately follow the SD model. Moreover, the transport of *p*-xylene in PIM-1-derived CMS in **PIM-1 _Ar_500_CMS** and **PIM-1_4% H**_**2**_**_500_CMS** has also been shown to closely follow the SD model.^13,43^ Here, to study if the transport of water follows the SD model in PIM-1-derived CMS, the sorption coefficient () and the Maxwell–Stefan diffusivity (*Đ*) were derived from gravimetric vapor sorption experiments.

**Figure 5:**
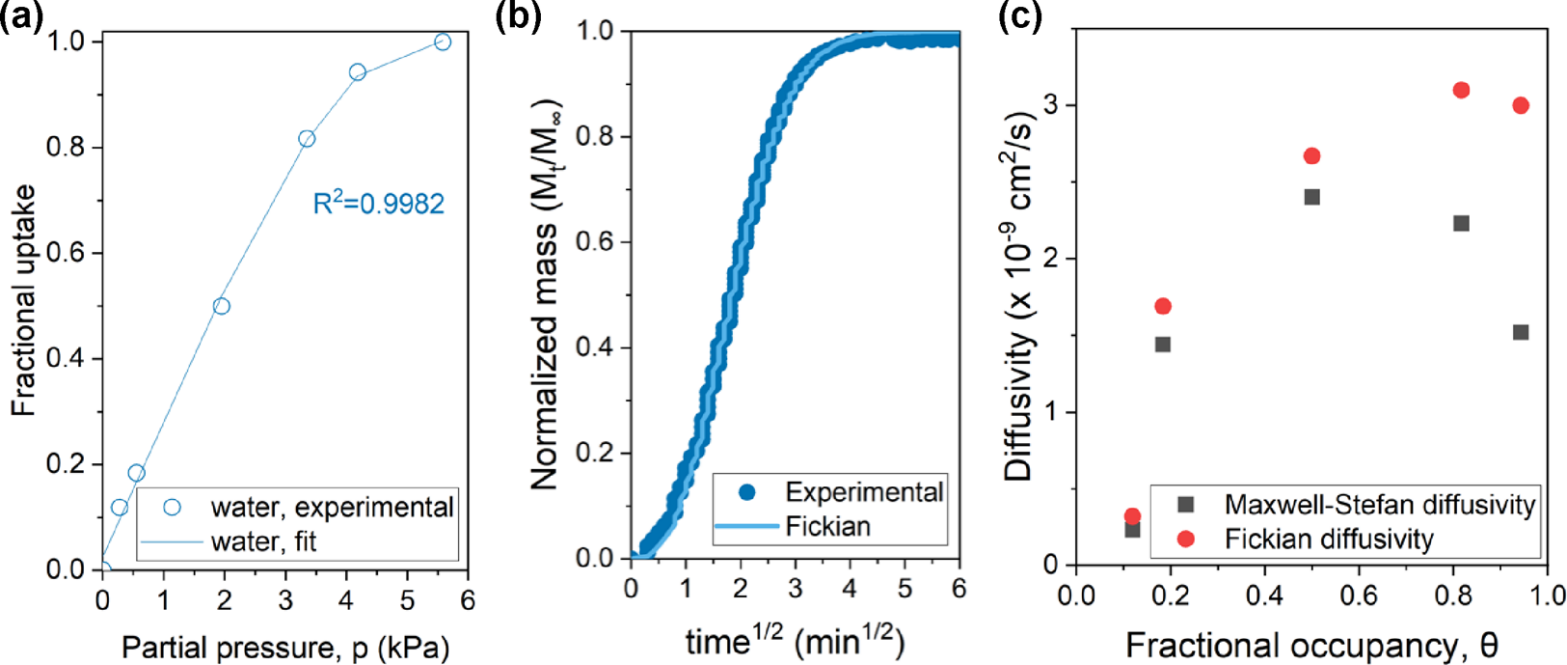
Sorption and diffusion properties of water in various CMS samples. (a) Vapor sorption isotherms of water on **PIM-1_4% H_2__500_CMS**. The experimental data are represented as circles, while the cubic polynomial modeled fit is depicted by the line plots. (b) Transient uptake curve of water on **PIM-1_4% H_2__500_CMS** at an activity of 0.6. (c) Maxwell–Stefan diffusivity and Fickian diffusivity of water on **PIM-1_4% H_2__500_CMS** with respect to the fractional occupancy of water in the film. All of the water uptakes were measured at 35 °C.

The transport properties of water in **PIM-1_4% H**_**2**_**_500_CMS** were experimentally obtained. The gravimetric water vapor sorption experiment was conducted on dense film **PIM-1_4% H**_**2**_**_500_CMS** samples (Figure 5). For simplicity, the water isotherm on **PIM-1_4% H**_**2**_**_500_CMS** was modeled using a cubic polynomial equation (eq (S8)). Using the modeled isotherm, the sorption coefficient of water in **PIM-1_4% H**_**2**_**_500_CMS** is derived using eq (S2). The experimental upstream and downstream activities obtained from the pure component WK vapor permeation ( = 0.85 and  = 0.21) were utilized as the boundary conditions in the estimation of the sorption coefficients. The sorption coefficient of water in **PIM-1_4% H**_**2**_**_500_CMS** was calculated as 2.8  (Table 1).

**Table 1:** Sorption-Diffusion Model Estimates of Water and *p*-Xylene Vapor Permeabilities in **PIM-1_4% H_2__500_CMS** and Comparison with Pure Component Experimental Permeabilities Obtained via WK

	***T***		*Đ*		
units	°C				
water	35	2.8	227.3	634.3 (1893.3)	2779.5 (8297.1)
*p*-xylene	35	1.2	5.4^a^	6.5 (19.4)	6.5 (19.4)

a Obtained from / = *Đ.*

The diffusion coefficient of water was obtained based on the kinetic sorption curve as in Figure 5b. The transport diffusivities were obtained by fitting the transient curves to the Fickian transport equation ( eq S7) at different sorbate loadings. The Fickian diffusivities were then thermodynamically corrected (eq 8) to obtain the MS diffusivities. The MS diffusivity at a water activity of 0.53 was used in the SD model calculation to represent the experimental conditions in the WK permeation apparatus (i.e., the average of the upstream and downstream activities of the membranes). The MS diffusivity at an activity of 0.53 was interpolated from the MS diffusivity at activities of 0.35 and 0.6 (Figure 5a,b). This is a current limitation of our work as we do not have a full understanding of the guest loading gradient within the membrane. Thus, we resort to using MS diffusivity at a fractional occupancy that we feel is representative when averaged over the length of the membrane, assuming that the guest loading gradient is linear across the membrane. The diffusion coefficients, *Đ*, of water in **PIM-1_4% H**_**2**_**_500_CMS** were derived as . The diffusion coefficient of water in **PIM-1_4% H**_**2**_**_500_CMS** is comparable to that in **PVDF_Ar_500_CMS**, .^22^ While this might be initially surprising, our PSD analysis suggests the presence of significant volumes of >3.4 Å limiting ultramicropores in both samples and water (kinetic diameter = 2.65 Å) is sufficiently small enough that it is largely insensitive to these minor changes in the limiting ultramicropore dimensions.

**Figure 6:**
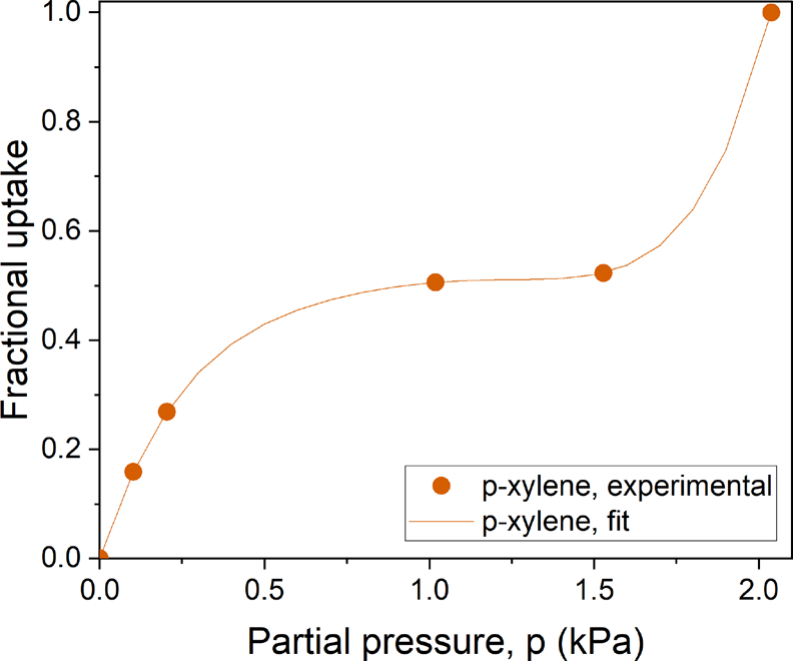
Vapor sorption isotherms of *p*-xylene on **PIM-1_4% H_2__500_CMS** at 35 °C. The experimental data are represented by the circle points, while the fifth-degree polynomial fit is depicted by the line plot. The *p*-xylene uptake at unit activity was obtained from a liquid water-soaking experiment, assuming that the solvent-accessible pores by water are fully occupied by the liquid *p*-xylene adsorbates.

The gravimetric vapor sorption experiment was also conducted with *p*-xylene on **PIM-1_4% H**_**2**_**_500_CMS** (Figure 4 and Figure 6). In Figure 6, the *p*-xylene isotherm on **PIM-1_4% H**_**2**_**_500_CMS** is fit with a fifth-degree polynomial equation (eq (S9)). The experimental upstream and downstream activities obtained from the pure component WK vapor permeation ( = 0.85 and  = 0.00) were also utilized in the estimation of the *p*-xylene sorption coefficients. The sorption coefficient of *p*-xylene is also obtained using eq (S2) as 1.2 . As the uptake of *p*-xylene into our CMS films was too slow to reliably measure using gravimetric vapor sorption techniques, we obtained the diffusivity of *p*-xylene by back-calculating from the experimental permeabilities and sorption coefficients (i.e., / = *Đ*). The thermodynamically corrected diffusion coefficient of *p*-xylene in **PIM-1_4% H**_**2**_**_500_CMS** is estimated to be  (Table 1).

As shown in Table 1, the Maxwell–Stefan sorption-diffusion model was able to reasonably estimate the permeability of water ( within a factor of 4 of the experimental permeability (). The water permeability prediction was 4-fold smaller than that of the experimental permeability, where the difference could perhaps be the result of some small defective pathways in the CMS membranes in permeation experiments; similar findings were observed in the case of the PVDF CMS.^22^ The diffusion coefficients, *Đ*, of water and *p*-xylene in **PIM-1_4% H**_**2**_**_500_CMS** were estimated to be  and , respectively, resulting in the diffusion selectivity of water over *p*-xylene of 42. Moreover, the sorption selectivity of water over *p*-xylene was found to be 2.5.

### 4.3 Water and *p*-Xylene Vapor Permeation in Various CMS Membranes

Vapor permeation of water and *p*-xylene in various CMS membranes was measured in a WK vapor permeation system. Both pure and mixture permeation experiments were conducted at three temperatures (38, 45, and 55 °C), and the saturator temperature was fixed at 35 °C. The mixture vapor stream was thus generated with a *p*-xylene partial pressure of 2.05 kPa and a water partial pressure of 4.77 kPa. The thickness of each membrane was measured after the permeation experiments (Table S 4) and was utilized in the permeability calculation. This work includes permeation experiments on the PIM-1-derived CMS membranes, and the **PVDF_Ar_500_CMS** permeation data are excerpted from ref (22).

**Figure 7:**
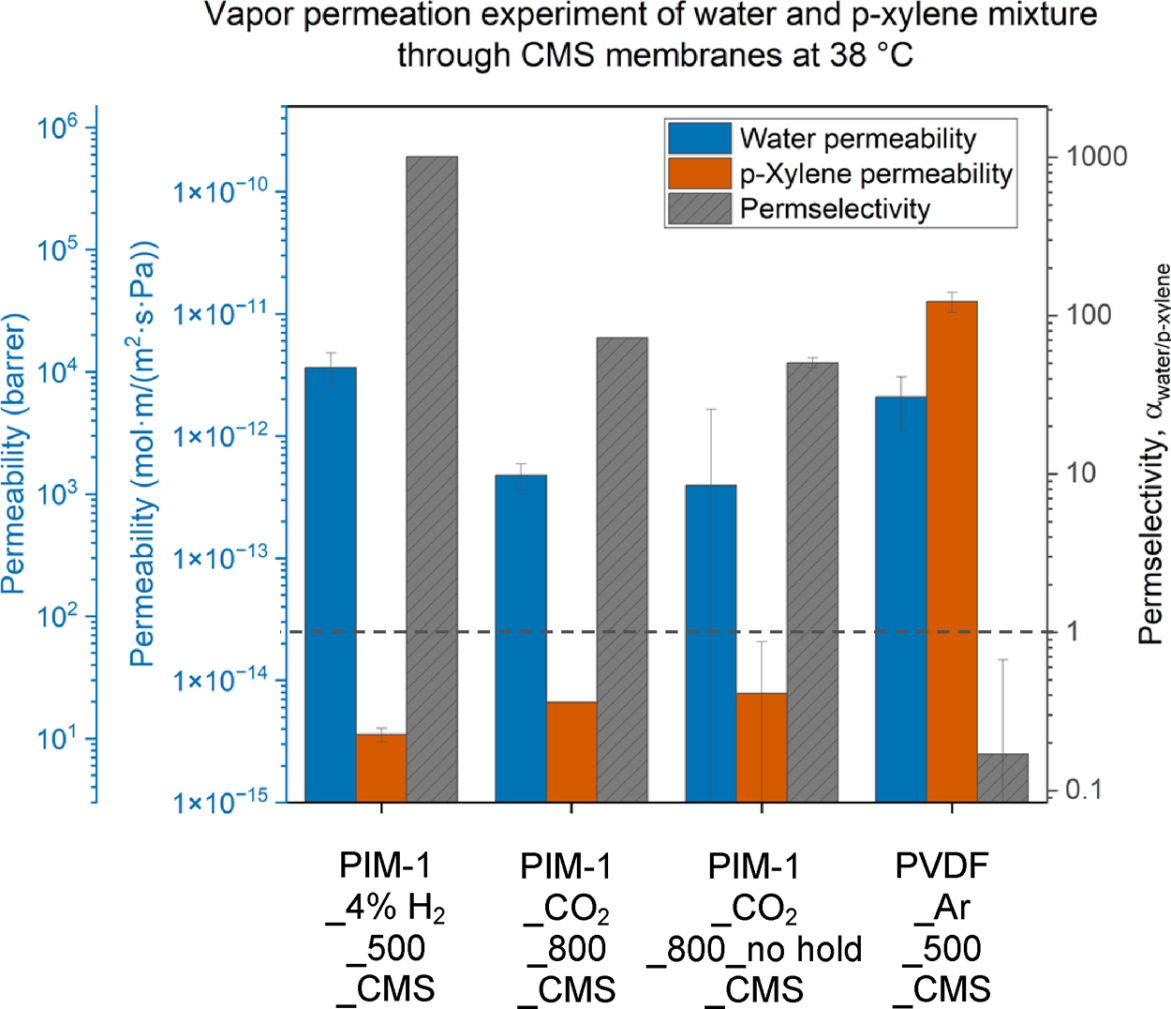
Vapor permeation experiment of water and *p*-xylene mixtures through various CMS membranes at 38 °C. Water activity upstream = 0.72 ± 0.01, *p*-xylene activity upstream = 0.81 ± 0.09, film thickness = 21.1 ± 1.8 μm, helium feed flow rate = 23.4 sccm, and helium sweep flow rate = 4.7 sccm for the experiments on the PIM-1-derived CMS. PVDF-derived CMS permeation measurement is excerpted from ref (22).

The mixture permeation of water and *p*-xylene vapors at 38 °C is provided in Figure 7. Comparing **PVDF_Ar_500_CMS** to other PIM-1 derived CMS, **PVDF_Ar_500_CMS** shows an organic-selective separation, where α_water/*p*-xylene_ < 1, while the PIM-1-derived CMS shows water-selective separation (α_water/*p*-xylene_ > 1). This is mainly attributed to the low water sorption affinity observed in **PVDF_Ar_500_CMS** (Figure 4a). Moreover, as studied in ref (22), the diffusion-selective contribution to water–*p*-xylene separation in **PVDF_Ar_500_CMS** is not sufficiently large (*Đ*_water_/*Đ*_*p*-xylene_ ∼ 1.5 at 35 °C) to overcome the strong sorption selectivity of *p*-xylene ( ∼ 4.2 at 35 °C). On the other hand, the PIM-1-derived CMS membranes are selective toward water permeation by exhibiting mixture permselectivities (α_water/*p*-xylene_) of 1010, 72, and 50 from **PIM-1_4% H**_**2**_**_500_CMS**, **PIM-1_CO**_**2**_**_800_CMS**, and **PIM-1_CO**_**2**_**_800_no hold_CMS**, respectively.

The high permselectivity in water-selective separation can be attributed to both the sorption and diffusion selectivity in PIM-1-derived CMS. As shown in the SD transport study in **PIM-1_4% H**_**2**_**_500_CMS**, the diffusion coefficient of water in **PIM-1_4% H**_**2**_**_500_CMS** is comparable to that in **PVDF_Ar_500_CMS,** which is reported to be .^22^ On the other hand, the *Đ* of *p*-xylene in **PIM-1_4% H**_**2**_**_500_CMS** is ∼35 times slower than that in **PVDF_Ar_500_CMS,** which is reported as .^22^ In addition to being slightly sorption-selective toward water over *p*-xylene (2.5 at 35 °C), **PIM-1_4% H**_**2**_**_500_CMS** is significantly diffusion-selective toward water (*Đ*_water_/*Đ*_*p-*xylene_ ∼ 42 at 35 °C), resulting in a water-selective separation in the water and *p*-xylene mixture, in contrast to **PVDF_Ar_500_CMS**, which exhibited a *p*-xylene-selective separation due to stronger sorption selectivity.

We also observe that the separation performance of the PIM-1-based CMS materials could be varied dramatically by varying the pyrolysis conditions. A decrease in water permeability is observed in the order **PIM-1_4% H**_**2**_**_500_CMS**, **PIM-1_CO**_**2**_**_800_CMS**, and **PIM-1_CO**_**2**_**_800_no hold_CMS**. High water permeability in **PIM-1_4% H**_**2**_**_500_CMS** is attributed to the high sorption affinity for water, which is seen in the vapor sorption isotherm in Figure 4a,b. Both the CO_2_-pyrolyzed CMS samples exhibit suppressed water uptake in the low activity region, which suggests weaker interactions between water and the CMS surface. These weaker interactions ultimately result in reduced water permeability due to smaller sorption coefficients (Figure 7). Moreover, the CO_2_-pyrolyzed CMS with no hold exhibits slightly reduced water permeability, which we attributed to insufficient formation of micropores.

These differences in water permeability and *p*-xylene permeability led to an increase in α_water/*p*-xylene_ in the order of **PIM-1_CO**_**2**_**_800_no hold_CMS**, **PIM-1_CO**_**2**_**_800_CMS**, and **PIM-1_4% H**_**2**_**_500_CMS**. The sample PIM-1 pyrolyzed in H_2_ (**PIM-1_4% H**_**2**_**_500_CMS**) showed the highest water permeability and the lowest *p*-xylene permeability, leading to the highest mixture α_water/*p*-xylene_ of 1010 at 38 °C. Such high separation performance is attributed to the narrow distribution of ultramicropores in this material (Figure 3). Moreover, in the mixture permeation of water and *p*-xylene, water and *p*-xylene molecules would compete for access and interaction with the membrane pores. The relatively hydrophilic characteristic of **PIM-1_4% H**_**2**_**_500_CMS** would lead to competitive sorption of water, and the condensed water clusters in pores could potentially hinder the diffusion of *p*-xylene.^44,45^ The α_water/*p*-xylene_ of the samples pyrolyzed in CO_2_ (**PIM-1_CO**_**2**_**_800_CMS**) is significantly reduced to a value of 72, primarily due to significant increases in the *p*-xylene permeability and decreases in water permeability, likely due to decreases in the water sorption affinity. The transport pathway would be more accessible for *p*-xylene in **PIM-1_CO**_**2**_**_800_CMS** where pores are less blocked by water.^44,45^ Moreover, we speculate that the *p*-xylene permeability increases due to the broader ultramicropore and micropore distributions in this material. Removing the pyrolysis soak (**PIM-1_CO**_**2**_**_800_no hold_CMS**) further reduces the permselectivity to 54, and this is tentatively assigned to the insufficient organization of graphenic nanoribbons. The kinetic restriction that contributes to selective transport is mainly controlled by the slit-shaped ultramicropores, not by the larger ultramicropores formed in the continuous phase by the orphan strands.^28^ Therefore, it is hypothesized that the removal of the hold step results in the insufficient organization of the graphenic nanoribbons, causing CMS to be composed of more orphan strands than the organized platelet-like structures with graphenic slits, thus contributing to reduced molecular sieving separation.

The activation energy of permeation of pure and mixed water and *p*-xylene in each CMS material was investigated (Figure S 6) by using an Arrhenius equation (eq (S3)) and by measuring the permeabilities at three different temperatures (38, 45, and 55 °C). The activation energy of permeation in **PVDF_Ar_500_CMS** is also excerpted from ref (22). Figure S 6 shows that the pure water permeations in all CMS membranes show negative activation energies of permeation, identifying that the water transport in CMS is sorption-controlled. Also, pure *p*-xylene permeations in all CMS membranes show a positive activation energy of permeation, indicating that the transport of *p*-xylene in CMS is diffusion-controlled. The activation energies for permeation in pure and mixture permeation were then compared. The mixture permeation in PIM-1-derived CMSs shows the same sign activation energy for water and *p*-xylene as the pure component permeation, where the water transport is sorption-limited and the *p*-xylene transport is diffusion-limited. The dominance in sorption or diffusion in PIM-1-derived CMS remains the same as the mixture transport, which exhibits water-selective “molecular sieving”-style separation (see Section 5). This is contrary to the observation in the PVDF-derived CMS, which exhibits *p*-xylene selectivity in mixture permeation. In the PVDF-CMS case, the water permeation in the mixture shows a positive activation energy of permeation, representing diffusion-limited transport. The diffusion-limited transport of water in the *p*-xylene mixture can be attributed to the increased kinetic restriction of water as a result of the competitively sorbed *p*-xylene. Moreover, the *p*-xylene permeation in the mixture shows a negative activation energy of permeation, indicating sorption-limited transport. Such an observation corroborates the competitive sorption-driven separation of water and *p*-xylene, as it shows an increased contribution of *p*-xylene sorption to the *p*-xylene selective transport in the mixture separation.

### 4.4 Comparison of SD Model Estimates with Experimental Observations for Various Membrane Modalities

#### 4.4.1 Water Transport Analysis in CMS Membranes in RO, PV, and VP Processes using the SD Model

**Figure 8:**
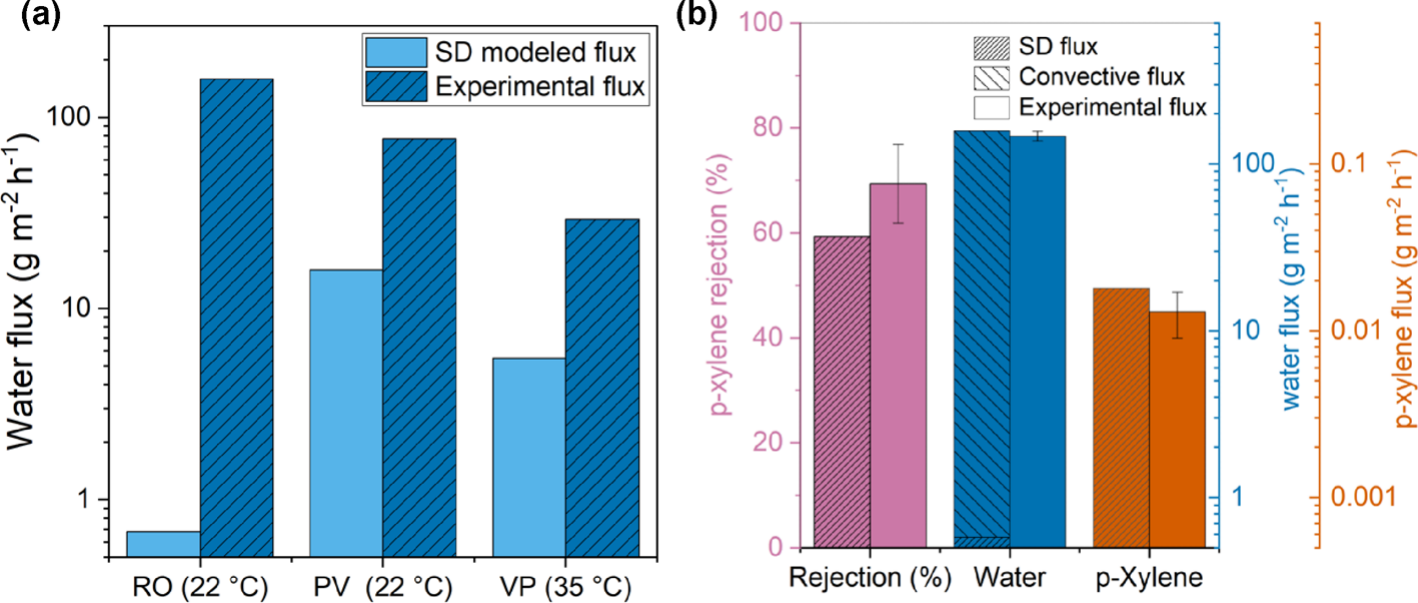
Comparisons between model and experiment. (a) Comparison of experimental water flux and SD model-calculated water flux in RO, PV, and VP modalities. (b) Comparison of experimental and SD model-calculated fluxes of water and dilute concentration (∼290 ppm) of dissolved *p*-xylene. RO systems are operated at a transmembrane pressure of 10 bar, and the PV system was operated at an atmospheric pressure with helium sweep on the membrane downstream at 18 sccm flow rate.

Experiments on pure liquid water transport in RO and PV systems were conducted to provide insights into the mechanisms governing water-based transport in CMS membranes. The **PIM-1_4% H**_**2**_**_500_CMS** membranes, which exhibited high selectivities for water over *p*-xylene in the vapor permeation experiments, were fabricated into an asymmetric hollow fiber membrane (Figure S 2) and further investigated in liquid separation modalities. The estimation of pure water fluxes in **PIM-1_4% H**_**2**_**_500_CMS** membranes in different membrane processes, RO, PV, and VP, was conducted and compared to the experimental observations (Figure 8a and Table S 5). The flux estimation was based on the assumption that the overall mass transfer resistance is governed by the mass transfer resistance in the selective separation layer of the asymmetric hollow fiber membranes. The parameters used for the flux estimation were obtained from gravimetric sorption and diffusion experiments on water vapor, and the boundary conditions were obtained from the RO, PV, and VP pure water permeation experiments. In the RO system, a transmembrane pressure of 10 bar was applied, and the PV system employed a He sweep (∼18 sccm) on the downstream side of the membrane. A detailed description of the model calculation and parameters is provided in the Supporting Information (S4 1)

The simplified SD expressions demonstrated reasonable predictions for the experimental fluxes in the isobaric experiments (i.e., PV and VP), with the PV and VP fluxes falling within a 5-fold difference from the experimental values. The experimental PV and VP fluxes were ∼5 times higher than the SD predicted fluxes, which could be a result of somewhat larger micropores or defects within the CMS hollow fiber membranes (see Section 5). The gas permeation experiment was conducted on the **PIM-1_4% H**_**2**_**_500_CMS** hollow fiber membrane modules after the modules had been used in liquid permeation experiments. The results of the gas permeation experiments indicate that the membranes did not exhibit detectable defects (details are provided in the Supporting Information (S4. 4)). The VP flux prediction using the simplified SD equation (eq 6) was able to show similar accuracy to the more robust prediction of VP shown in Table 1. Such a result supports the assumptions made in the simplified equation for water transport systems.

On the other hand, the SD prediction for the RO flux significantly underestimated the experimental flux by ∼250×. This observation can be attributed to defects or larger continuous micropores present in the CMS membranes. These defects—if present—will play a significant role in the flux within the pressurized RO system due to continuum-level Poiseuille flow. Such Poiseuille flow through these defects can be conservatively treated as a nonselective convective flux, which can be obtained using eq 10.
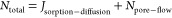
10

The pore size distribution of **PIM-1_4% H**_**2**_**_500_CMS** was investigated using cryogenic nitrogen physisorption^13^ (recreated in Figure S 4), and it has shown the presence of larger micropores above 12 Å. These larger micropores and the micropores at 5–7 Å (Figure 3) could potentially serve as defects in the membrane structure, allowing for Poiseuille flow of liquid water through these pore sizes exceeding 2–3 times the kinetic diameter of a water molecule (2.65 Å). These larger micropores could have originated from either the material pyrolysis process or the membrane fabrication process.

#### 4.4.2 Water–*p*-Xylene Mixture Transport Analysis in the CMS Membrane in the RO Process Using the SD Model

The co-transport of water with dilute concentrations of *p*-xylene in the RO system was explored using the SD model and comparisons to experimental permeation data. A feed mixture of ∼290 ppm concentration of *p*-xylene dissolved in water was separated in **PIM-1_4% H**_**2**_**_500_CMS** membranes in the RO system.

The isotherms of water and *p*-xylene in **PIM-1_4% H**_**2**_**_500_CMS** exhibit complex non-Langmuir isotherms (Figure 5a and Figure 6, respectively), making it challenging to model water and *p*-xylene in the Maxwell–Stefan formulation.^43,46,47^ As a result, in the calculation of diffusive flux (eq 6) of water and *p*-xylene in a mixture system, the average fractional occupancy of component *i* in the binary mixture system, , was obtained using a simplified estimation of sorption selectivity (eq 12).^23^ The sorption isotherm for water was modeled using the linear model of Henry’s law (eq 11), viz.,
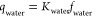
11where *q*_water_ (mmol/g) is the uptake of water in the membrane, *K*_water_ (mmol/g/kPa) is Henry’s constant for water in the membrane, and *f*_water_ is the water fugacity (kPa) of the bulk fluid phase on the upstream or the downstream of the membrane. This simplification facilitated the calculation of uptake under pressurized liquid conditions, where the fugacity exceeds the saturation fugacity, *f*^sat^ (which is simply the vapor pressure of the pure liquid). The use of Henry’s law is reasonable given the nearly linear uptake curve observed in the water isotherm on **PIM-1_4% H**_**2**_**_500_CMS** (Figure S 5a, *R*^2^ = 0.978). The *p*-xylene isotherm exhibited a complex isotherm shape, which we modeled using a fifth-degree polynomial (eq (S9)) for simplicity.

The calculation of fractional uptake in a binary adsorbent system is simplified and expressed in eq 12, utilizing single-component isotherms. It was assumed that the fractional occupancy on membrane pores is 1 (i.e.,  +  ∼ 1) when in contact with a liquid phase.
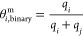
12

Detailed parameters for the mixture modeling can be found in Table S 6. The permeate mole fraction is calculated using permeate molar fluxes (eq 13). The separation performance in the RO system was expressed in terms of the separation factor (β_*i*/*j*_) (eq 9).
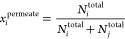
13

The reverse osmosis water and *p*-xylene separation in **PIM-1_4% H**_**2**_**_500_CMS** was experimentally performed, showing a ∼69.4% rejection of *p*-xylene (Figure 8 and Table S 6) and a water flux of 147.0 L m^–2^ h^–1^. This experimental flux for water and *p*-xylene was then compared with the estimates from the sorption-diffusion model. As shown in the pure water permeation experiments, the SD model was unable to replicate the experimental water fluxes in the hydraulic permeation case. We assign the difference between the experimental fluxes and SD fluxes as the “convective flux”. This convective flux was then used to estimate the water flux in the mixture permeation experiments along with the SD model flux (i.e.,  =  + , where  is the convective flux from the single component permeation experiment). The flux estimates for both *p*-xylene and water closely aligned with the experimental fluxes (when the “convective flux” of water through the defects is considered by using the pure water fluxes under hydraulic permeation conditions, Table S 5), with an error of within 5%. The convective flux of water is assumed to be the same in the pure water and water–*p*-xylene mixture experiments, as the water mole fraction in the feed is near 1 in the dilute concentration of *p*-xylene dissolved in aqueous solution, and both pure and mixture systems are applied with the same 10 bar pressure gradient. Additionally, the separation factor of water/*p*-xylene was accurately estimated with an error of 25%.

#### 4.4.3 Water and *p*-Xylene Separation in the Pervaporation System

The simplified SD model was also employed to estimate the co-transport of water and *p*-xylene in CMS membranes in PV systems,^48^ and it presents insights into the effect of sweep gas on the membrane downstream. Similar to the RO estimates, the diffusive fluxes of water and *p*-xylene were calculated by using eq 6, and the fractional occupancy in the binary mixture system, , was obtained by using eq 12. Polynomial fittings are employed to model the water and *p*-xylene isotherms (eqs (S8) and (S9), respectively).

Detailed parameters for the mixture permeation modeling in PV can be found in Table S 7. The fractional occupancies in the downstream membrane pores are assumed to be 0 (i.e.,  +  ∼ 0), assuming that the downstream permeate fugacities in the PV system are near zero. In the pervaporation experiment with pure water, the fractional occupancy downstream was nearly 1, as indicated in Table S 5, with an average value of θ̅^m^ = 0.99. This high fractional occupancy was due to the low experimental sweep gas flow rate. However, when dealing with mixture systems in pervaporation, we estimate the downstream fractional occupancy by assuming it to be 0, as our understanding of mixture fractional occupancy is limited when  +  is not equal to 1. The comparisons of the SD model fluxes with the experimental fluxes for the water and *p*-xylene pervaporation separation using **PIM-1_4% H**_**2**_**_500_CMS** are shown in Table S 8. The PV prediction suggests that an effective concentration of *p*-xylene on the permeate due to the high activity coefficient of infinitely dilute *p*-xylene in water should be observed. Moreover, the effect of dilution of the permeates on the separation performance was investigated. When the permeates were minimally diluted by the sweep gas, the fluxes of water and *p*-xylene were low due to a small fugacity gradient driving force while exhibiting an effective concentration of *p*-xylene on the permeate. On the other hand, when the permeates were significantly diluted, both water and *p*-xylene fluxes were maximized. However, the separation factor of *p*-xylene/water decreases due to the higher maximum fugacity gradient of water in the presence of a dilute concentration of organic solvents in the aqueous feed. The flow rate of sweep gas can be adjusted to control the separation performance of water and the dilute concentration of *p*-xylene in pervaporation.

## 5 Discussion

**Figure 9:**
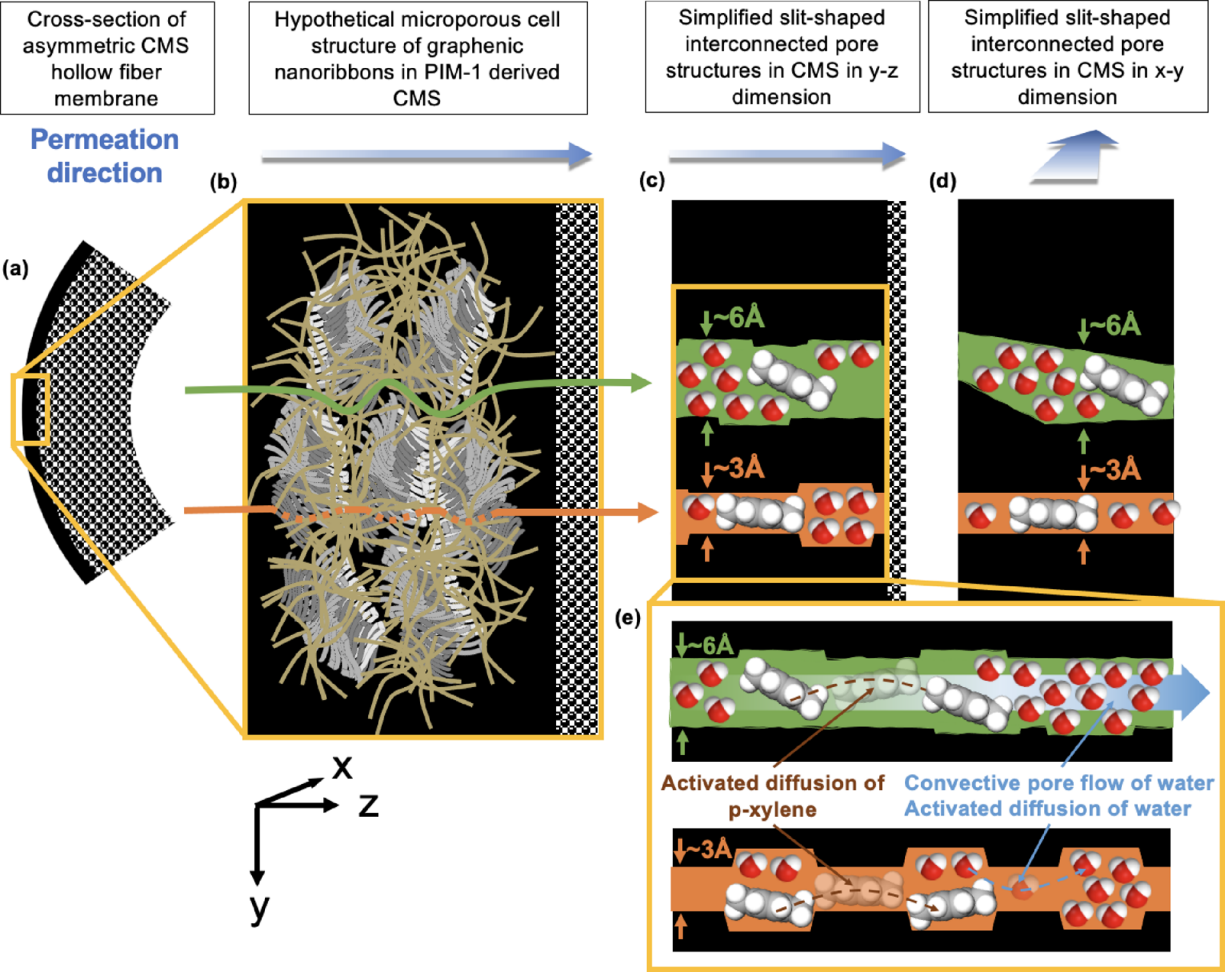
Hypothetical schematic of water and *p*-xylene cotransport in **PIM-1_4% H_2__500_CMS** in a hydraulic permeation experiment. (a) Cross-section of the asymmetric hollow fiber CMS membrane. The transport of water and *p*-xylene occurs from the shell side to the bore side. (b) Magnified schematic of the selective layer of the asymmetric hollow fiber CMS membrane. The hypothetical structure of **PIM-1_4% H_2__500_CMS** is shown, which consists of a microporous cell matrix composed of graphenic plates with ultramicroporous gaps. The microporous cell matrix is surrounded by a network of orphan strands. (c) Simplified schematic of the slit-shaped microporous structure of **PIM-1_4% H_2__500_CMS**. The transport pathways exist in parallel through less-selective ultramicropores (5–7 Å) and more selective ultramicropores (3–4 Å). (d) Simplified schematic of the slit-shaped microporous structure of **PIM-1_4% H_2__500_CMS**, showing the dimension where the permeate is transporting into the plane of the page. The convective transport of water in 5–7 Å pores may occur around the *p*-xylene molecules in this direction, which are transported only via activated diffusion in the slit-shaped pores without blocking the pathway for water transport. Hypothetical structure of **PIM-1_4% H_2__500_CMS** reproduced or adapted with permission from ref (29). Copyright 2023 Elsevier.

The permeation of water–organic mixtures such as water/*p*-xylene through amorphous, permanently microporous structures such as carbon molecular sieves is clearly a complex process. Taken holistically, our combined data sets focusing on surface and textural analysis, diffusion and sorption coefficient measurements, and permeation measurements under different sets of driving forces suggest that there is a fertile ground for engineering CMS materials to enable different types of transport mechanisms. A common observation in VP, PV, and RO was that the predicted diffusive flux of water underestimated the experimental fluxes. This discrepancy was more evident in the pressurized RO system (∼250× for RO as compared to ∼4× for VP and PV). Such a difference in fluxes for the RO case (compared with the relatively good agreement in the VP and PV cases) suggests to us that water is permeating through the H_2_-pyrolyzed PIM-1 membranes via a Poiseuille-style mechanism. A potential impact of this observation is that it is likely possible to intentionally design CMS membranes such that the solvent molecule (water, in our case) permeates following a Poiseuille flow-style mechanism, which is important from a practical perspective as this transport mechanism can conceptually lead to high water fluxes. However, Poiseuille transport is often undesirable in practice, as the solute molecules are often nonselectively convected through the membrane. We show here that *p*-xylene in the **PIM-1_4% H**_**2**_**_500_CMS** sample transports only via a sorption-diffusion mechanism (i.e., via activated diffusive “hops” through the membrane ultramicropores), whereas water can transport through the same micropores—or perhaps through separate micropores not accessible to *p*-xylene—via a Poiseuille-style mechanism (Figure 9). CMS membranes ideally consist of microporous cell structures composed of carbon plates with ultramicroporous slits, which result in interconnected ultramicropores and micropores that exhibit a narrow distribution of pore sizes. Within this distribution, we speculate that there are interconnected pores that are continuous through the membrane that facilitate both diffusive fluxes of water and *p*-xylene (with an ultramicropore size of 3–4 Å). Additionally, we speculate that there exists a population of continuous pores in the 5–7 Å range, which are potentially created in the continuous phase of orphan strands. These larger and less selective ultramicropores may serve as a convective pathway for water while still acting as a diffusive pathway for *p*-xylene (Figure 9). In these slit-shaped pores, the convective transport of water may occur around the *p*-xylene molecules transporting via activated diffusion. Although this is a speculative interpretation, the resulting differences in the SD transport rate of *p*-xylene and the pore flow transport rate of water in such a situation provide interesting combinations of solute rejection and water flux.

Importantly, these same membranes can also be used in pervaporation applications in which the transmembrane pressure is minimized relative to the RO, thus allowing the membranes to operate predominantly in an SD mode for all penetrants (i.e., water and *p*-xylene). From the vapor permeation experiments, we find that the PIM-1 CMS membranes are permselective for water, while the PVDF CMS membranes are permselective for *p*-xylene, likely due to differences in the water sorption isotherms in the two carbon membranes. Nevertheless, the high activity coefficient of *p*-xylene in the feed mixture allows for significant enrichment of *p*-xylene in the membrane permeate for both classes of membranes, suggesting an interesting path forward for both water purification and solute recovery applications via the combination of RO and PV in a membrane cascade.

## 6 Conclusions

Here, the transport of water and *p*-xylene in CMS membranes was studied with a focus on various factors that might affect the transport rates. These include surface chemistry, pore size distribution, diffusion and sorption coefficients, and different membrane separation modalities. CMS membranes were fabricated using different polymer precursors, pyrolysis atmospheres, final pyrolysis temperatures (*T*_p_), and pyrolysis temperature profiles. The mixture transport of water and a dilute concentration of dissolved *p*-xylene in the RO system was calculated and compared with the experimental data. The RO separation experiments exhibited a *p*-xylene rejection of ∼69.4% under 10 bar transmembrane pressure gradient with a water permeance of 147.0 L m^–2^ h^–1^ bar^–1^. In contrast to the significant convective flux observed in water transport, the flux of the dilute concentration of *p*-xylene did not exhibit a convective component, as its flux was predicted by the SD calculations. When the convective flux of water was accounted for, the mixture flux was reasonably well estimated as was the *p*-xylene rejection (∼59.3%). The mixture transport prediction of water and a dilute concentration of dissolved *p*-xylene was also investigated in the PV system. Contrary to the RO separation, the PV prediction exhibited an effective concentration of *p*-xylene on the permeate due to the high activity coefficient of infinitely dilute *p*-xylene in water.

When the RO and PV data are inspected in the context of our single component sorption, diffusion, and permeation data, we hypothesize the presence of larger, interconnected micropores that enable the Poiseuille-style transport of water while inhibiting such transport for *p*-xylene. Such a combination of pore flow and solution-diffusion fluxes for the solvent and solute, respectively, is a potential path forward for engineering high-performance membranes with high water permeance and solute rejections.

It is important to acknowledge the limitations of this study. Assumptions were made in the SD model to simplify the flux calculations, including the utilization of a linear uptake model in the pressurized liquid system, a simplified mixture uptake approach, and the assumption of zero total uptake downstream in pervaporation. Consequently, although the findings provide valuable insights into transport under different scenarios, they may not encompass the entirety of the phenomenon. Indeed, key questions exist regarding the nature of the water transport mechanism in these permanently microporous materials. Nevertheless, we believe that these findings offer valuable insights into the engineering conditions necessary to achieve the desired separation performance of a membrane in a given separation modality, whether it involves the rejection or the concentration of *p*-xylene in the permeate stream.

## Supporting Information Available

Details on the transport theory and background, methods, materials, and the transport predictions of water and *p*-xylene in CMS membranes in various modalities (PDF)
