# Correction for “Structure–Transport Relationships of Water–Organic Solvent Co-transport in Carbon Molecular Sieve (CMS) Membranes”

**DOI:** 10.1021/acs.iecr.5c01842

**Published:** 2025-05-28

**Authors:** Young Hee Yoon, Yi Ren, Akriti Sarswat, Suhyun Kim, Ryan P. Lively

There were two important typos identified in the reported units of water flux under reverse osmosis conditions. In Section 4.4.2 (Water-p-Xylene Mixture Transport Analysis in the CMS membrane in the RO Process using the SD model) of the Results and Discussion, the water flux was originally stated as 147.0 L m^–2^ h^–1^ and should be corrected to 147.0 g m^–2^ h^–1^. Additionally, in the Conclusions, the water flux had a similar typo, and was originally reported as 147 L m^–2^ h^–1^ bar^–1^ and should be revised to 147 g m^–2^ h^–1^.

The original datasets and tables all have the correct units, and the conclusions of the research are based around the correct units, and as such there are no changes to the conclusion of the manuscript. We submit this correction to avoid future confusion around the water fluxes through the membrane.

