# Supplementary material for: Structure–Transport Relationships of Water–Organic Solvent Co-transport in Carbon Molecular Sieve (CMS) Membranes
Source: Ind Eng Chem Res. 2023 Oct 30;62(44):18647–61. doi: 10.1021/acs.iecr.3c02519 (PMC10636745; doi:10.1021/acs.iecr.3c02519)
Supplement: Supplementary file 1 — ie3c02519_si_001.pdf [file ie3c02519_si_001.pdf]

Supplementary information for:

Structure-transport relationships of water-organic solvent co-transport in carbon molecular sieve (CMS) membranes

Young Hee Yoon[a], Yi Ren[a], Akriti Sarswat[a], Suhyun Kim[a], and Ryan P. Lively*[a]

[a] School of Chemical and Biomolecular Engineering, Georgia Institute of Technology, Atlanta, GA 30332

*Corresponding Authors: ryan.lively@chbe.gatech.edu

Table of Contents

S 1. Theory and background 2

S 2. Methods and materials 4

S2. 1. Materials 4

S2. 2. Synthesis of PIM-1 5

S2. 3. Fabrication of CMS membranes 5

S2. 4. Material characterization 8

S2. 5. Vapor sorption measurements 9

S 3. Supplementary data for CMS material characterization and vapor permeation study 11

S 4. Transport prediction of water and p-xylene in CMS membranes in various modalities 14

S4. 1. Water transport analysis in CMS membranes in RO, PV, and VP process using the SD model........ 14

S4. 2. Water-p-xylene mixture transport analysis in CMS membrane in the RO process using the SD model........ 17

S4. 3. Water and p-xylene separation prediction in the pervaporation system 18

S4. 4. Gas permeation experiment on PIM-1_4% H_2_500_CMS hollow fiber membranes after the liquid permeation experiments to check for defects. 22

References 24

S 1. Theory and background

The SD model is expressed in Fickian (or "transport") diffusivity, D, as the model is derived based on Fick's law of diffusion [1]. However, the Fickian diffusivity varies depending on the guest molecule loading. Therefore, this study employs a Maxwell-Stefan (MS) diffusivity ($Đ$) that ideally remains independent of guest loading (Eq. (S1)). Here, an expression for the sorption coefficient, $\mathbb{S}$, (Eq. (S2)) is derived from the SD model and the Maxwell-Stefan mass transfer formulation for single component transport. The permeability, $\mathbb{P}$, can be determined via the normalized flux (Eq. (1)) as well as via the sorption-diffusion model (Eq. (3)), which offers an experimental method to evaluate the applicability of the SD model using several distinct experiments (e.g., sorption isotherms, kinetic uptakes of guest molecules, and steady-state permeation of guest molecules). The flux can be expressed using the MS formulation [2] in terms of the CMS density, ρ_{CMS}, MS diffusivity, $Đ$, the thermodynamic correction factor, Γ, the saturation loading, q^{sat}, and the fractional loading in the membrane, θ. ρ_{CMS} is approximated as 2.0 g/cm^3 [3,4]. The remaining expression for permeability besides the MS diffusivity becomes an expression for sorption coefficient $\mathbb{S}$.

$$\mathbb{P} = \frac{N \cdot \ell}{\Delta f} = Đ \cdot \frac{(\rho_{\mathrm{CMS}} \Gamma q^{\mathrm{sat}} \nabla\theta) \cdot \ell}{\Delta f} = Đ \cdot \mathbb{S} \tag{S1}$$

$$\mathbb{S} = \frac{(\rho \Gamma q^{\mathrm{sat}} \nabla\theta) \cdot \ell}{\Delta f} \tag{S2}$$

In permeation that follows the SD transport model, the activation energy for permeation can be obtained from an Arrhenius relationship that describes the temperature dependence of the permeabilities (Eq. (S3)) where $\mathbb{P}_{o,\mathrm{A}}$ is a pre-exponential factor, $E_{\mathbb{P},\mathrm{A}}$ is an activation energy for permeation, R is the universal gas constant, and T is the absolute temperature. A deeper

understanding of how the penetrants transport can be gained by analyzing the temperature-dependent sorption and diffusion coefficients, which respectively represent the kinetic and thermodynamic aspects of permeability. The temperature dependence of the sorption coefficient and the diffusion coefficient can be described by a van't Hoff expression (Eq. (S4)) and an Arrhenius relationship (Eq.(S5)), respectively. $\mathbb{S}_{o,\mathrm{A}}$ is a pre-exponential factor, $\Delta H_{\mathbb{S},\mathrm{A}}$ is an integral heat of sorption, $Đ_{o,\mathrm{A}}$ is a pre-exponential factor, and $E_{Đ,\mathrm{A}}$ is an activation energy for diffusion. Combining Eq. (3) and (S3)-(S5), the activation energy of permeation can be expressed as the sum of the activation energy of diffusion and heat of sorption (Eq. (S6)). $E_{Đ,\mathrm{A}}$ is always positive as the diffusion coefficient increases with the temperature increase. The activation energy for diffusion describes the energy required for a penetrant to make a diffusive jump from one sorption state to another. $\Delta H_{\mathbb{S},\mathrm{A}}$ is generally negative for an exothermic adsorption process. When the negative $\Delta H_{\mathbb{S},\mathrm{A}}$outweighs the positive $E_{Đ,\mathrm{A}}$, $E_{\mathbb{P},\mathrm{A}}$ is negative, indicating that the membrane permeability coefficient decreases with increasing temperature and vice versa. Therefore, by examining the $E_{\mathbb{P},\mathrm{A}}$, we can understand whether sorption or diffusion dominates transport.

$$\mathbb{P}_{\mathrm{A}} = \mathbb{P}_{o,\mathrm{A}} \exp\left(\frac{-E_{\mathbb{P},\mathrm{A}}}{RT}\right) \tag{S3}$$

$$\mathbb{S}_{\mathrm{A}} = \mathbb{S}_{o,\mathrm{A}} \exp\left(\frac{-\Delta H_{\mathbb{S},\mathrm{A}}}{RT}\right) \tag{S4}$$

$$Đ_{\mathrm{A}} = Đ_{o,\mathrm{A}} \exp\left(\frac{-E_{Đ,\mathrm{A}}}{RT}\right) \tag{S5}$$

$$E_{\mathbb{P},\mathrm{A}} = E_{Đ,\mathrm{A}} + \Delta H_{\mathbb{S},\mathrm{A}} \tag{S6}$$

The simplified diffusive flux expression (Eq. (6)) is derived based on two key assumptions: 1) a linear fugacity gradient, and 2) a constant average guest loading across the membrane. These

assumptions enable the application of the simplified flux equation in membrane systems involving pressurized liquid streams, such as in RO. For instance, the estimation of guest molecule loading in a pressurized liquid environment (where the fugacity is > f^{sat}) is not feasible using vapor sorption isotherm. Hence, assuming a constant average guest loading across the membrane with a linear fugacity gradient facilitates the estimation of the guest loading even in a pressurized liquid environment.

S 2. Methods and materials

S2. 1. Materials

Tetrafluoroterephthalonitrile (TFTPN) (Sung-Young Chemical Limited, Shanghai, China) and 5,5'6,6'-tetrahydroxy-3,3,3',3'-tetramethyl-1,1'-spirobisindane (TTSBI) (Alfa Aesar) were purified before use for PIM-1 synthesis. TFTPN was recrystallized using vacuum sublimation at 140 °C. TTSBI was purified via reprecipitation from hot methanol with dichloromethane. Poly(vinylidene fluoride) (PVDF) was purchased from Alfa Aesar. The synthesized PIM-1 polymer and commercially purchased PVDF polymer were dried in a vacuum oven overnight at 110 °C and 60 °C, respectively, before use.

Methanol (CH_3OH, 99%), potassium carbonate (K_2CO_3, anhydrous, 99%), chloroform ($CHCl_3$, >99.8%), dichloromethane (DCM, >99.5%), dimethylformamide (DMF, >99.8%), tetrahydrofuran (THF, >99.5%), p-xylene (99 %) were purchased from Alfa Aesar and were used as received. N,N-Dimethylacetamide (DMAc, anhydrous 98 %, Sigma-Aldrich), methanol (MeOH, anhydrous, 99.8 %, Sigma-Aldrich), p-xylylenediamine (99.5 %, TCI America), and sodium hydroxide (NaOH, ACS reagent, ≥ 97.0 %, pellets, Sigma-Aldrich) were used as received. DI water was provided in lab via an Elga DV35 Purelab Option water purification system.

Argon (UHP 5.0 Grade), 4 vol% hydrogen balanced with argon, carbon dioxide (Bone dry), and helium (UHP grade) were purchased from Airgas.

S2. 2. Synthesis of PIM-1

The PIM-1 was synthesized using the low-temperature polycondensation technique developed by Budd et al. [5]. The purified TFTPN and TTSBI monomers were dissolved in a 1:1 molar ratio in anhydrous DMF. Finely ground anhydrous K_2CO_3 was added to the solution in a 2.5 mol equivalent amount of TFTPN to initiate the polymerization reaction. The reactants were continuously stirred for 72 hours under nitrogen at 65 ^oC. The reaction mixture was then allowed to cool to room temperature and was quenched in DI water to end the reaction and precipitate the PIM-1 polymer. Fluorescent yellow PIM-1 was recovered from the solution via vacuum filtration, washed with additional DI to remove excess salts, and purified with chloroform to obtain a high molecular weight polymer. The molecular weight was determined by gel permeation chromatography (GPC) in HPLC grade chloroform. The PIM-1 used for film membrane fabrication was determined to be M_w = 157k with a *PDI* = 5.8, and the PIM-1 for hollow fiber membrane fabrication is determined to be M_w = 39k with a *PDI* = 3.4 when compared against polystyrene standards.

S2. 3. Fabrication of CMS membranes

Dense PIM-1 film membranes were prepared in a solvent-saturated bag to decrease the solvent evaporation rate from the dense film and create defect-free dense membranes. The casting bag was saturated with THF for 6 hours. 7 ml of 2 wt% PIM-1 dissolved in THF was poured into the Teflon petri dish. Once the film membranes were dried in the casting bag, the membranes were removed and dried in a vacuum oven at 80 ^oC.

Dense PVDF film membranes were also cast in a solvent-saturated bag. The casting bag was saturated with DMAc for two days. 25 wt% PVDF in DMAc solution was cast on a glass plate with an 8ML casting blade. Once the membranes were fully solidified, the films were transferred to a vacuum oven for total DMAc evaporation at 60 °C overnight.

PIM-1 asymmetric hollow fiber membranes were fabricated using a dual-bath method in dry-wet spinning, using the methods developed [6]. The PIM-1 hollow fiber membranes were fabricated by co-extruding three solution layers, bore fluid, polymer dope, and sheath solution. The polymer dope comprised PIM-1, solvent, and non-solvents (15 wt% PIM-1, 69.5 wt% THF, 13.25 wt% DMAc, and 2.25 wt% ethanol). The bore fluid was composed of solvent and non-solvents (45 wt% THF, 46.75 wt% DMAc, and 8.25 wt% ethanol) to keep the center of the fibers hollow. The sheath solution (82.5 wt% 1-butanol and 17.5 wt% THF) protected against too-fast evaporation of the volatile solvent THF and creates a defect-free skin layer. The spinning was conducted under the lab temperature of 20.5 ºC and the relative humidity of 10 %. The bore, core, and sheath solutions were pumped through the spinneret using mechanical syringe pumps (1000D for sheath and core, 500D for bore fluid, Teledyne Isco) at a flow rate of 90, 105, and 65 ml/hour. The three-layer extrusion through the triple orifice spinneret was sent to the DI-water quench bath after an air gap of 1.5 cm. The extrusion went through phase inversion in the quench bath with a bath temperature of 50 ºC. The phase-inverted polymer fiber was guided through the quench bath and collected on a take-up drum at a 1.5 m/min speed. The fibers were rotated on the take-up drum for 1 hour to ensure sufficient phase inversion. The fibers were then collected on a separate DI water bath for complete phase inversion. The fibers were solvent exchanged in DI water for 3 days and were repeated 3 times. The fibers were solvent exchanged with less surface tension solvents such as methanol for 2 days for 3 times and then hexane for 2 days for 3 times, to protect the

porous structure of the fiber. Then the fibers were air-dried and were activated in a vacuum oven at 80 ºC before use.

The precursor membranes were pyrolyzed in a 3-zone furnace, illustrated in Figure S 1. The detailed pyrolysis protocol for PIM-1 and PVDF precursors is provided in Table S 1.

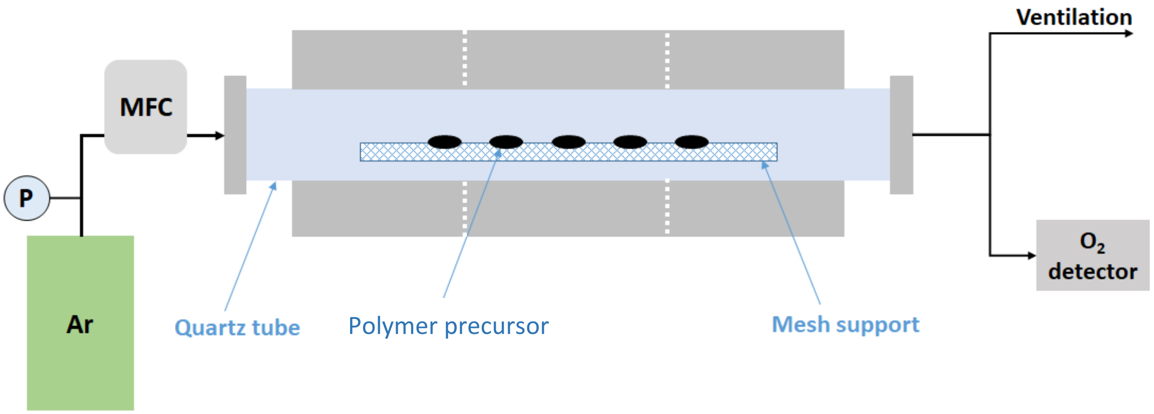

Figure S 1. Schematic diagram of pyrolysis furnace set-up for CMS fabrication. The diagram is drawn for fabrication of film membranes supported on a stainless-steel mesh, but the hollow fiber membranes were pyrolyzed similarly. The diagram is drawn for argon pyrolysis, but the same setup was used for other gas pyrolysis conditions.

Table S 1. Pyrolysis temperature thermal profiles for PVDF [3] **and PIM-1 derived CMS**

	**PVDF-CMS**		**PIM-1 CMS**		
**Phase**	**Heating rate (°C/min)**	**Final pyrolysis temperature**	**Heating rate (°C/min)**	**Final pyrolysis temperature**	
		**500 °C**		**500 °C**	**800 °C**
Ramp	10	50-250 °C	10	18-100 °C	18-100 °C
Activate	-*	-	0	at 2 hours at 100 °C	
Ramp	3.8	250-485 °C	10	100-200 °C	100-500 °C
	0.25	485-500 °C	3	200-485 °C	500-785 °C
	-	-	0.25	485-500 °C	785-800 °C
Soak	0	at 2 hours at 500 °C	0	at 2 hours at 500 °C	at 2 hours at 800 °C
Cool	-	Natural cooling back to room temperature under pyrolysis environment	-	Natural cooling back to room temerature under pyrolysis environment	

*ex-situ activation before pyrolysis

The PIM-1 hollow fiber membranes pyrolyzed into **PIM-1_4% H_2_500_CMS** asymmetric hollow fiber membranes are shown in Figure S 2.

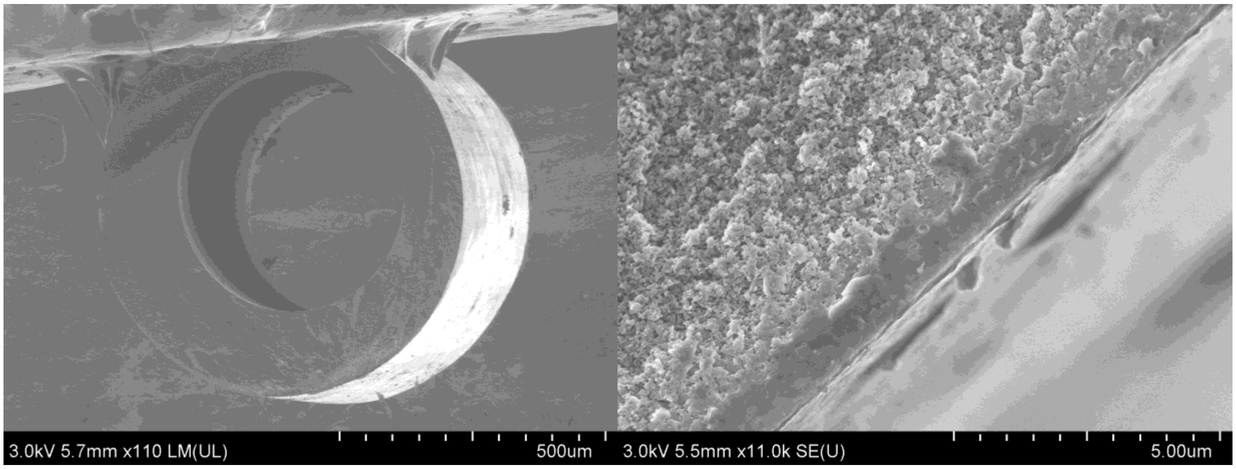

Figure S 2. SEM images of PIM-1_4% H_2_500_CMS asymmetric hollow fiber membranes. The left image shows the whole cross-section of the hollow fiber membrane, and the right image shows the selective layer located on the shell side of the asymmetric membrane.

S2. 4. Material characterization

X-ray photoelectron spectroscopy (XPS) was conducted using a K-Alpha XPS (Thermo Fisher Scientific, West Palm Beach, FL). The instrument is equipped with a monochromatic Al-Kα X-ray source. The spectra were calibrated by the internal standard using silver oxide. Before collecting the XPS spectra, the XPS analysis chamber was evacuated to 2×10^{-8}mbar or lower pressure. The XPS data were collected from CMS powder samples, which are ground from dense CMS films. Therefore, the collected chemical composition should represent the average composition of the membrane material.

Scanning electron microscopy (SEM) micrographs were obtained on a Hitachi SU 8210 FE-SEM microscope at 3-5 kV beam energy.

Carbon dioxide physisorption on PIM-1-derived CMS and PVDF-derived CMS was measured at 273 K in ASAP 2020 HD (Micromeretics, USA). The carbon dioxide isotherm was measured in 0.1-760 torr range. The analysis temperature was controlled using an ice water bath. The pore size distributions were calculated based on the CO_2 isotherms using the HS-2D-NLDFT (heterogeneous surface two-dimensional non-local density functional theory) model provided in the MicroActive software package (Micromeritics, USA) [7].

S2. 5. Vapor sorption measurements

For the gravimetric vapor sorption experiment, the CMS film membranes were cut into small dimensions of around 0.5 cm by 0.5 cm to fit in the sample boat. The effective membrane area for the sorption experiments was ~6 cm^2, with around 16 μg of CMS membrane samples for each experiment. Before each sorption experiment, the samples were dried in situ at 110 ºC for 2000 min under flowing nitrogen. The equilibrium criteria for each step were set to mass changes less than 0.0005 wt% for 15 minutes or manually via adjusting the maximum run time until the uptake reaches equilibrium.

Water and p-xylene diffusion behavior in CMS was examined by measuring the transient mass change in the **PIM-1_4% H_2_500_CMS** dense film membrane at a set relative humidity/pressure. Thick and dense film samples with an average thickness of ~25 μm were used to capture the guest transport inside the membrane as the controlling mass transfer resistance. The thickness of the membranes was measured in SEM. Ten cross-sections of the membranes were measured and averaged to obtain the membrane thickness. The transient mass uptake data were normalized and fitted to a Fickian mass transfer equation to obtain the transport diffusion coefficient, D. When using the Fickian solutions to analyze the transient uptake data, it is important

to consider the exponential boundary condition because the vapor concentration in the sample chamber does not change instantaneously. Eq. (S7) is a Fickian mass transfer equation derived by Crank [8] for diffusion in sheet (film) membranes with an exponential boundary condition in the bulk concentration. M_t is the total mass of CMS and the adsorbed vapor at time t, and M_∞ is the total mass of CMS and the equilibrium amount of adsorbed vapor at given relative humidity/pressure. β is a time constant from the exponential boundary conditions. D is a transport diffusion coefficient, and ℓ is half the membrane thickness. The transport diffusivity and the time constant were determined by fitting experimentally measured kinetic uptake curves.

$$\frac{M_t}{M_\infty} = 1 - \exp(-\beta t)\left(\frac{D}{\beta\ell^2}\right)^{\frac{1}{2}} \tan\left(\frac{\beta\ell^2}{D}\right)^{\frac{1}{2}} - \frac{8}{\pi^2}\sum_{n=0}^{\infty} \frac{\exp\left(-\frac{(2n+1)^2\pi^2 Dt}{4\ell^2}\right)}{(2n+1)^2\left[1-(2n+1)^2\left\{\frac{D\pi^2}{(4\beta\ell^2)}\right\}\right]} \tag{S7}$$

Moreover, the kinetic uptake curves were assessed by two criteria to ensure to represent of the isothermal and internal diffusion resistance: 1) Ruthven-Lee's criteria [9] to ensure the vapor sorption was isothermal, and 2) evaluate whether the exponential boundary condition or the guest diffusion in the membrane is the controlling resistance [10]. The exponential boundary condition in the concentration change can be quantified by the external time constant β (s^{-1}), obtained in Eq. (S7). The length-normalized external time constant and the diffusion coefficient was calculated, $\phi = \frac{\ell^2\beta}{D}$. Measurements with $\phi > 5$ were considered to represent the internal diffusion resistance with an acceptable margin of error [10] and were used to calculate the diffusion coefficients.

S 3. Supplementary data for CMS material characterization and vapor permeation study

Table S 2. Micropore volumes of various CMS materials measured from CO_2 isotherms at 273K. The CO_2 isotherms are measured up to $P/P^{sat} < 0.03$, which has uptakes in pore sizes < 10 Å.

CMS membranes	Micropore volume (cm^3/g stp) < 10 Å
**PIM-1_Ar_500_CMS**	46.3
**PIM-1_4% H_2_500_CMS**	49.1
**PIM-1_CO_2_500_CMS**	50.6
**PIM-1_CO_2_800_CMS**	71.9
**PIM-1_CO_2_no hold_800_CMS**	66.0
**PVDF_Ar_500_CMS**	64.6
**PVDF_CO_2_500_CMS**	67.4

Table S 3. Pore size distribution of various CMS materials in the microporous region < 10 Å using CO_2 isotherms at 273K and 2D-NLDFT.

Pore Width (Å)	Pore Volume, dV/dW (cm^3/g·Å)						
	**PIM-1_ Ar_ 500_ CMS**	**PIM-1_ 4% H_2_ 500_ CMS**	**PIM-1_ CO_2_ 500_ CMS**	**PIM-1_ CO_2_ 800_ CMS**	**PIM-1_ CO_2_no hold_ 800_ CMS**	**PVDF_ Ar_ 500_ CMS**	**PVDF_ CO_2_ 500_ CM**
3-4 Å	0.167	0.175	0.166	0.134	0.132	0.161	0.148
4-5 Å	0.150	0.151	0.158	0.370	0.364	0.192	0.239
5-6 Å	0.081	0.095	0.120	0.092	0.071	0.129	0.111
6-7 Å	0.122	0.129	0.124	0.244	0.215	0.188	0.215
7-8 Å	0	0	0.037	0.010	0.005	0.067	0.059
8-9 Å	0	0	0	0.010	0	0.014	0.045
9-10 Å	0	0	0	0	0	0	0

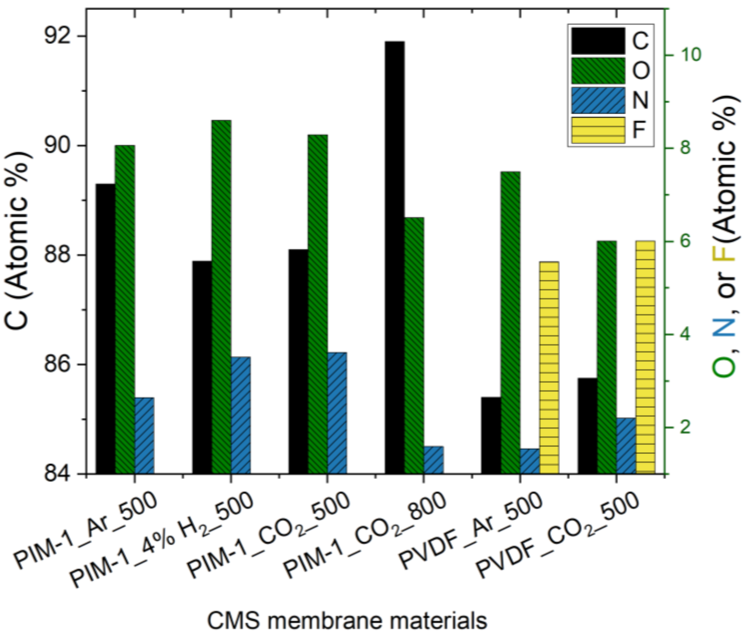

Figure S 3. Carbon, oxygen, nitrogen, and fluorine atomic composition in PIM-1- and PVDF-derived CMS powders obtained from XPS Survey scans.

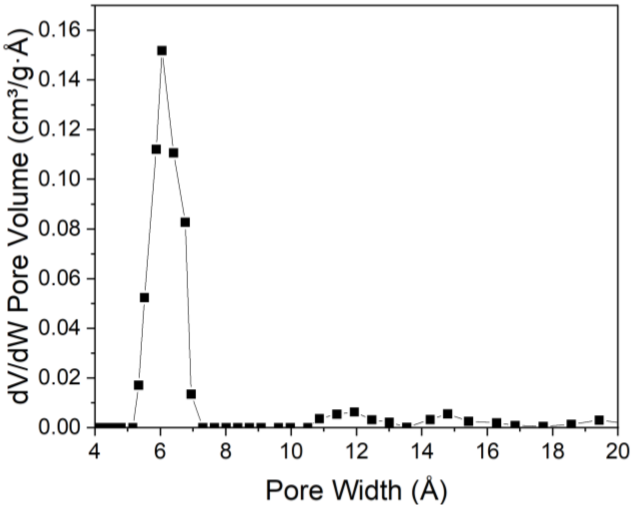

Figure S 4. Pore size distribution of PIM-1_4% H_2_500C_CMS obtained from cryogenic N_2 physisorption at 77K. The physisorption data are provided from Ma et al. [11]**, and the pore size distribution is calculated using HS-2D-NLDFT (MicroActive software, Micromeritics, USA).**

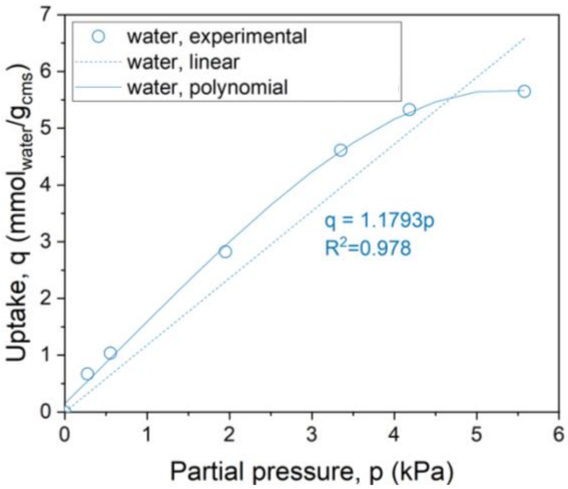

Figure S 5. The vapor uptake isotherm of water in PIM-1_4% H_2_500_CMS. The data points represented by circles are obtained from the experimental vapor sorption isotherm presented in Figure 5a. The dashed line represents the linear model employed in the liquid transport study for estimating water uptake, and the R^2 is provided for the linear model fitting. The solid lines depict the polynomial models fitted, adapted from Figure 5a.

The water and p-xylene isotherms on **PIM-1_4% H_2_500_CMS** were modeled using Eq. (S8) and (S9), respectively.

$$q_{water} = a \cdot f_{water}{}^{3} + b \cdot f_{water}{}^{2} + c \cdot f_{water} + d \quad \text{(S8)}$$

q is an uptake on CMS (mmol/g_{CMS}), and f is the fugacity (kPa). $a, b, c,$ and d are cubic polynomial fitting constants for the water isotherm.

$$q_{\text{p-xylene}} = a \cdot f_{\text{p-xylene}}{}^{5} + b \cdot f_{\text{p-xylene}}{}^{4} + c \cdot f_{\text{p-xylene}}{}^{3} + d \cdot f_{\text{p-xylene}}{}^{2} + e \cdot f_{\text{p-xylene}} + f \quad \text{(S9)}$$

a, b, c, d, e and f are fifth degree polynomial fitting constants for p-xylene isotherm.

Table S 4. Average membrane thickness measured for the dense CMS membranes in WK vapor permeation experiments

**CMS membranes**	Thickness (µm)
**PIM-1_4% H_2_500_CMS** (this work)	23.0 ± 5.7
**PIM-1_CO_2_800_CMS** (this work)	19.3 ± 1.2
**PIM-1_CO_2_no hold_800_CMS** (this work)	20.9 ± 1.0
**PVDF_Ar_500_CMS** [12]	17.5

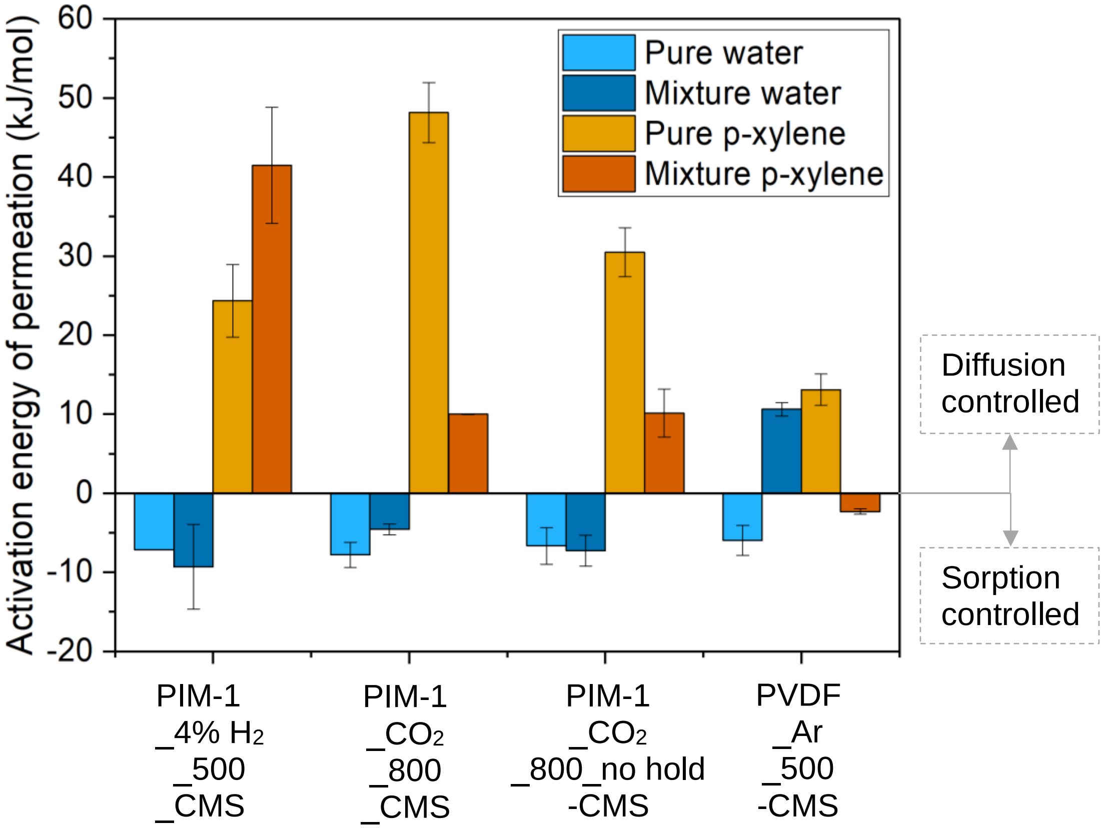

Figure S 6. The activation energy for permeation of water and p-xylene at pure and mixture permeation in various CMS membranes

S 4. Transport prediction of water and p-xylene in CMS membranes in various modalities

S4. 1. Water transport analysis in CMS membranes in RO, PV, and VP process using the SD model.

The MS diffusivity of water in **PIM-1_4% H_2_500_CMS** obtained from vapor isotherm shows a dependency on guest molecule loading (Figure 5c). Therefore, the estimation of $Đ^{m}_{water}$

for RO, PV, and VP involved considering each average fractional loading, $\bar{\theta}_{\mathrm{water}}^{\mathrm{m}}$, based on the experimental upstream and downstream fugacities.

The average fractional occupancy of water, $\bar{\theta}_{\mathrm{water}}^{\mathrm{m}}$, for the RO system was estimated to be 1, considering that the fractional occupancy in both the upstream and downstream would be close to 1 when exposed to a liquid phase. The $\bar{\theta}_{\mathrm{water}}^{\mathrm{m}}$ for the PV and VP systems were estimated to be 0.99 and 0.63, respectively, determined based on the experimental measurements of the upstream and downstream activities. It is worth noting that despite both PV and VP having sweep gas on the membrane downstream, the estimated $\bar{\theta}_{\mathrm{water}}^{\mathrm{m}}$ value for PV is relatively high, closer to that of RO than VP. This can be attributed to the high water activity observed on the downstream of the membrane in the experiment, which is influenced by the low sweep gas flow rate employed (~18 sccm) and the fast water flux. The $\bar{\theta}_{\mathrm{water}}^{\mathrm{m}}$ estimations are based on the assumption of a linear guest loading profile in the membrane.

The MS diffusivity for the calculation of RO and PV flux, which experimentally exhibited $\bar{\theta}_{\mathrm{water}}^{\mathrm{m}}$ of 1 and 0.99, was determined at fractional occupancy θ = 0.94, measured at a relative humidity of 75 %. This value was limited by the capabilities of the gravimetric vapor sorption instrument (VTI SA+, TA instrument) used. The diffusivity for the VP system was obtained by interpolation at θ = 0.63. Furthermore, the sorption uptake at a unit activity, q_i^{sat}, was determined through a liquid water-soaking experiment.

It is noteworthy that the diffusivity and sorption parameters were determined at 35 °C, while the RO and PV permeation experiments were conducted at room temperature (~22 °C) as a result of current experimental capabilities (the sorption and diffusion analyzers require somewhat higher temperatures for consistent readings, and the permeation systems have not been outfitted

for safe operation with heating elements). The SD-modeled fluxes were first calculated at 35 °C for RO, PV, and VP systems. Subsequently, the SD calculated fluxes of RO and PV were adjusted to the flux at 22 °C utilizing the activated energy for water permeation in **PIM-1_4% H_2_500_CMS**, $\Delta E_{\mathbb{P}}$ = -7.2 kJ/mol (Figure S 6).

Table S 5. Parameters used for the calculation of the water flux in RO, PV, and VP systems using the SD model and the comparison to the experimental fluxes.

		**RO**	**PV**	**VP**
SD calculation	T_{SD} (°C)	35	35	35
	$Đ^{m}$ (cm^2/s)	1.52×10^{-9}	1.52×10^{-9}	2.27×10^{-9}
	ρ (g/cm^3)	2	2	2
	q^{sat} (mmol/g)	5.65	5.65	5.65
	$\bar{\theta}^{m}$	1	0.99	0.63
	$p^{upstream}$ (bar)	11	1	1
	$p^{downstream}$ (bar)	1	1	1
	$f^{upstream}$ (bar)	0.0562	0.0558	0.0476
	$f^{downstream}$ (bar)	0.0558	0.0472	0.0118
	$f^{average}$ (bar)	0.0560	0.0515	0.0297
	ℓ (cm)	0.00013	0.00013	0.0023
	$Flux^{SD}$ ($g/m^2/h$) at 35 °C	0.60	14.1	**5.5**
	$Flux^{SD}$ ($g/m^2/h$) at 22 °C	**0.68**	**15.9**	-
Experimental data	$T^{Experimental}$ (°C)	22	22	35
	$Flux^{Experimental}$ ($g/m^2/h$)	**158.5**	**76.9**	**29.3**
($Flux^{Experimental}$)/($Flux^{SD}$)		**231.8**	**4.8**	**5.3**
$Flux^{Convective}$ ($g/m^2/h$) at 22 °C, Δp= 10 bar		**157.9**	-	-

S4. 2. Water-p-xylene mixture transport analysis in CMS membrane in the RO process using the SD model

Table S 6. Detailed parameters used in SD model estimation of water-p-xylene mixture transport in PIM-1_4% H_2_500_CMS RO system.

RO		p-Xylene	Water
Parameters	$Đ^{m}$ (cm^2/s)	5.42×10^{-11}	1.52×10^{-9}
	ρ_{CMS} (g/cm^3)	2	2
	q^{sat} (mmol/g)	0.828	5.649
	x^{feed} (mole fraction)	4.73×10^{-5}	0.99995
	γ^{feed} (UNIQUAC) at 11 bar	5686	1
	$\gamma^{permeate}$ (UNIQUAC) at 1 bar	11848	1
	Δp (bar)	10	10
	f^{feed} (kPa)	0.52	5.61
	$f^{permeate}$ (kPa)	0.42	5.58
	Δf (kPa)	0.10	0.04
	Liquid mass fraction<br>in liquid saturated membrane	0.115	
	Henry's constant, K (mmol/g/kPa)	-	1.18
	Polynomial model parameter, a	0.28	-
	Polynomial model parameter, b	-1.34	-
	Polynomial model parameter, c	2.57	-
	Polynomial model parameter, d	-2.63	-
	Polynomial model parameter, e	1.53	-
	Polynomial model parameter, f	3×10^{-10}	-
	$\theta^{m,upstream}$	0.052	0.948
	$\theta^{m,downstream}$	0.048	0.952
	$\bar{\theta}^{m}$ (average)	0.050	0.950
	ℓ (cm)	0.00013	0.00013
Calculation Results	Diffusive flux (g/m^2/h) at **35 °C**	0.03	0.51
	Diffusive flux (g/m^2/h) at **22 °C**	0.02	0.58
	Convective flux (g/m^2/h) at **22 °C**	-	157.9
	**Total flux (g/m^2/h) at 22 °C**	**0.018**	**158.4**
	$x^{permeate}$ (mole fraction)	1.93×10^{-5}	0.99998

	separation factor water/p-xylene	2.5	
	p-xylene rejection (%)	59.3	
Experimental Data	**Flux (g/m^2/h) at 22 °C**	0.013 ± 0.004	147.0 ± 10.0
	separation factor water/p-xylene	3.1 ± 0.1	
	p-xylene rejection (%)	69.4 ± 7.5	

S4. 3. Water and p-xylene separation prediction in the pervaporation system

Initially, arbitrary values were assigned to the permeate mole fraction, which were used to calculate the downstream fugacities. The upstream and downstream fugacities were then used to calculate the Δf_i^{m} and $\bar{f}_i^{\mathrm{m}}$, allowing the calculation of the diffusive fluxes (Eq. (6)). Then the calculated diffusive fluxes were used to determine the permeate mole fractions, $x_{i,\mathrm{permeate}}$ (Eq. (13)). The calculated permeate mole fraction was subsequently used to calculate the downstream fugacities, and through iterations, the final concentrations for the permeate mole fraction were obtained where the input and output permeate mole fractions converged.

Table S 7. Detailed parameters used in SD model estimation for water-p-xylene mixture transport in pervaporation PIM-1_4% H_2_500_CMS

RO		p-Xylene	Water
Parameters	$Đ^m$ (cm^2/s)	5.42×10^{-11}	1.52×10^{-9}
	ρ_{CMS} (g/cm^3)	2	2
	q_{sat} (mmol/g)	0.828	5.649
	x_{feed} (mole fraction)	4.73×10^{-5}	0.99995
	γ_{feed} (UNIQUAC) at 1 bar	11848	1
	$\gamma_{permeate}$ (UNIQUAC) at 1 bar	11848	1
	P_{feed} (bar)	1	1
	f_{feed} (kPa)	0.52	5.61
	Liquid mass fraction in liquid saturated membrane	0.115	
	Polynomial model parameter, a	0.28	-0.02
	Polynomial model parameter, b	-1.34	0.05
	Polynomial model parameter, c	2.57	1.42
	Polynomial model parameter, d	-2.63	0.15
	Polynomial model parameter, e	1.53	-
	Polynomial model parameter, f	3×10^{-10}	-
	$\theta^{m}_{upstream}$	0.052	0.948
	ℓ (cm)	0.00013	0.00013

In pervaporation systems where a downstream sweep gas is used, the permeate mole fraction can be diluted by the sweep gas depending on the flow rate of the sweep gas (Figure S 7). Therefore, the dilution factors, k_{dilution}, as defined in Eq.(S10), were varied to investigate the effect of permeate dilution on the water-dissolved organic solvent separation. The diluted permeate mole fraction, $x_i^{\text{diluted permeate}}$, was used to calculate the downstream fugacity. k_{dilution} was varied in 0.01, 0.1, to 1 to represent conditions of high dilution to minimal dilution, respectively. The effect of the dilution factor on the separation parameters (flux, transmembrane fugacity, and separation factor) are presented in Figure S 8 and Table S 8. The pervaporation system employing

PIM-1_4% H_2_500_CMS for the separation of dissolved organics in aqueous solutions predicts the concentration of p-xylene in the permeate across various levels of permeate dilution.

$$x_i^{\text{diluted permeate}} = k_{\text{dilution}} x_i^{\text{permeate}} \tag{S10}$$

Under conditions of no dilution (k_{dilution}=1), where the sweep gas is used to carry the permeates to the condenser without diluting the permeate fugacities, the minimum fugacity gradients across the membrane are observed. As a result, both p-xylene and water exhibit the lowest fluxes. The separation factor of p-xylene/water was estimated to be ~559.

In contrast, under conditions of low downstream permeate activities, where the permeates are significantly diluted (k_{dilution}=0.01), both the p-xylene and water experience the maximum fugacity gradients across the membrane. This leads to an increase in both p-xylene and water fluxes. However, mainly due to the higher molar fraction of water in the feed stream, the feed water fugacity is higher than the feed p-xylene fugacity. Although both fugacity gradients reach their maximum values as the downstream fugacity approaches zero, the maximum fugacity gradient value is higher for water. Consequently, the increase in water fluxes is more pronounced, resulting in a lower separation factor of p-xylene over water. This relationship between flux and separation factor with respect to the dilution of permeate via sweep flow can be utilized, depending on the desired separation performance.

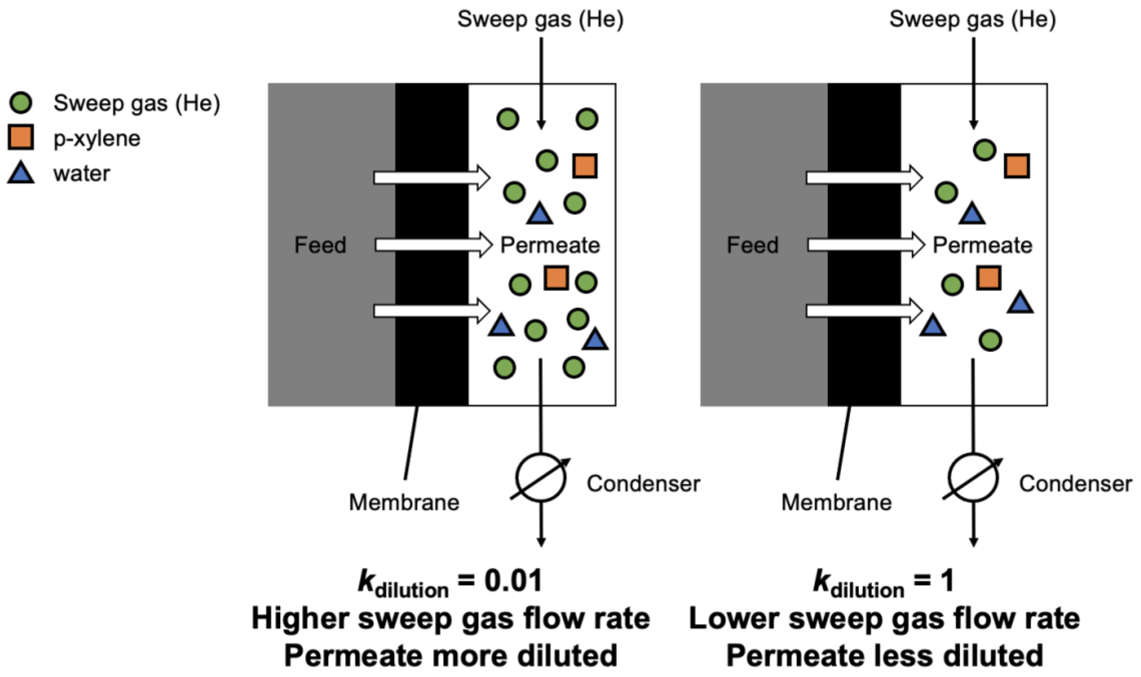

Figure S 7. A cartoon illustrating a pervaporation system, highlighting the varying levels of permeate dilution achieved through different sweep gas flow rates on the downstream side of the membrane. The numbers of sweep gas helium molecules, p-xylene molecules, and water molecules depicted in the diagram do not reflect the quantitative ratio between the molecules but serve as qualitative representations to demonstrate the differences between high dilution (e.g. $k_{dilution}$ = 0.01) and low dilution ($k_{dilution}$ = 1) conditions.

Table S 8. Calculation prediction of p-xylene and water separation in pervaporation using PIM-1_4% H_2_500_CMS.

**Dilution factor for permeates**	$x_{permeate, pX}$	$x_{permeate, w}$	Δf_{pX}	Δf_{w}	**$Flux_{pX}$**	**$Flux_{w}$**	**$SF_{pX/w}$**
-	Mole fraction	Mole fraction	kPa	kPa	$g/m^2/h$	$g/m^2/h$	-
**0.01**	4.0×10^{-4}	0.9996	1.0457	5.52	0.18184	78.2	8.3
**0.1**	4.7×10^{-4}	0.9995	1.0456	5.02	0.18181	65.3	10.0
**1**	2.6×10^{-2}	0.97	0.9979	0.15	0.16594	1.1	558.6

pX = p-xylene, w = water, SF = separation factor, α = permselectivity.

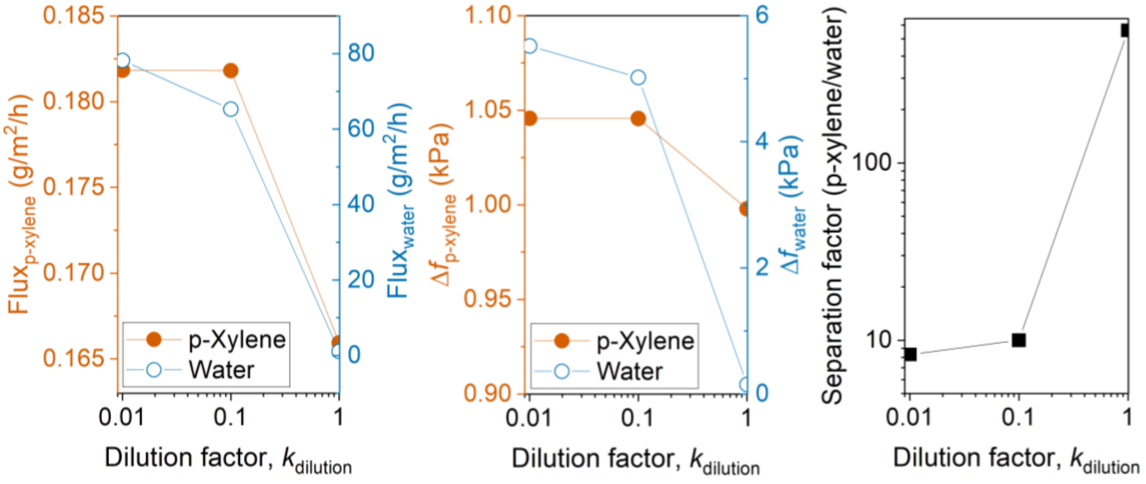

Figure S 8. a) Flux, b) transmembrane fugacity of p-xylene and water, and c) separation factor of p-xylene over water with respect to the dilation factor, $k_{dilation}$, for permeates in pervaporation separation of dilute p-xylene in an aqueous mixture.

S4. 4. Gas permeation experiment on PIM-1_4% H_2_500_CMS hollow fiber membranes after the liquid permeation experiments to check for defects.

Gas permeation experiments on **PIM-1_4% H_2_500_CMS** hollow fiber membranes were conducted to check for critical defects in the membranes. The membranes that were previously tested in pure liquid water and dilute concentration of p-xylene in aqueous mixture were then tested in nitrogen and helium permeation experiments. After the liquid permeation experiments, the membranes were immersed in aqueous solution of water and dilute concentration of p-xylene. These immersed membranes were prepared for the gas permeation tests by solvent exchange using methanol and hexane to enable gentle activation of the CMS membrane pores. The membrane modules were solvent exchanged with methanol three times and left overnight and the steps were repeated with hexane. Then the modules were air dried in air circulated hood overnight, then vacuum dried under 40 °C overnight.

The N_2 and He pure gas permeation experiments have been conducted at three pressure points (30, 60, and 80 psi) (Figure S 9) and positive yet decreasing slopes have been observed in flux in function of transmembrane pressure. This behavior is not unusual for sorption-diffusion membranes with penetrants that exhibit linear isotherms with the membrane, which is likely in the case of helium and nitrogen in CMS membranes. This flux profile yields a constant permeability as a function of transmembrane pressure, and importantly, reveals an ideal permselectivity for helium/nitrogen in excess of 7. This permselectivity value suggests that the membrane does not have detectable Knudsen-style defects.

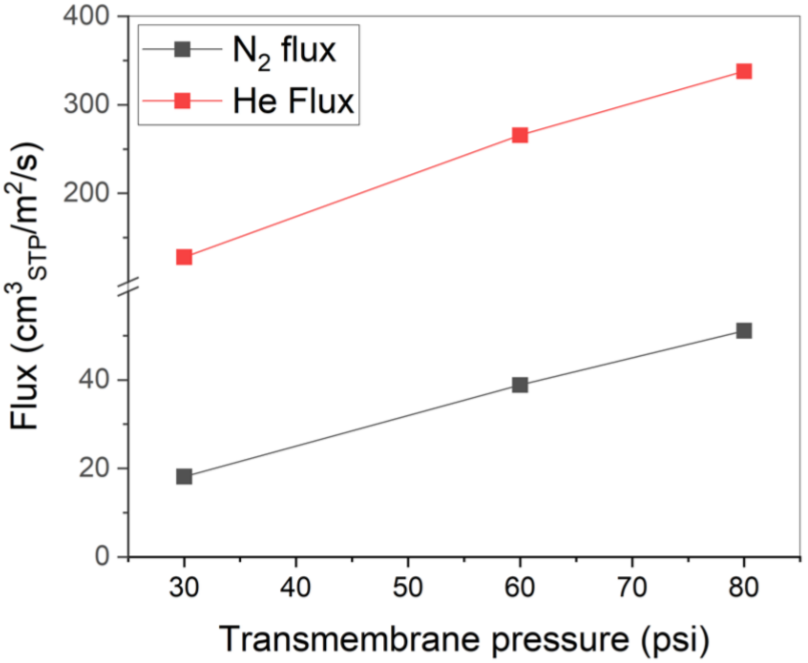

Figure S 9. Gas permeation experiment on PIM-1_4% H_2_500_CMS hollow fiber membranes after the liquid permeation experiments

References

[1] Paul, D.R., Reformulation of the solution-diffusion theory of reverse osmosis, *J. Membr. Sci*. **2004**, 241, 371–386.

[2] Krishna, R., Baur, R., Modelling issues in zeolite based separation processes, *Sep. Purif. Technol*. **2003**, 33, 213–254..

[3] Koh, D., McCool, B.A., Deckman, H.W., Lively, R.P., Reverse osmosis molecular differentiation of organic liquids using carbon molecular sieve membranes, *Science,* **2016**, 353, 6301, 808-807

[4] Ma, Y., Zhang, F., Yang, S., Lively, R.P., Evidence for entropic diffusion selection of xylene isomers in carbon molecular sieve membranes, *J. Membr. Sci*. **2018**, 564, 404–414.

[5] Budd, P.M., Elabas, E.S., Ghanem, B.S., Makhseed, S., McKeown, N.B., Msayib, K.J., Tattershall, C.E., Wang, D., Solution-Processed, Organophilic Membrane Derived from a Polymer of Intrinsic Microporosity, *Adv. Mater*. **2004**, 16, 456–459.

[6] Jue, M.L., Breedveld, V., Lively, R.P., Defect-free PIM-1 hollow fiber membranes, *J. Membr. Sci.*. **2017**, 530, 33–41.

[7] Jagiello, J., Ania, C., Parra, J.B., Cook, C., Dual gas analysis of microporous carbons using 2D-NLDFT heterogeneous surface model and combined adsorption data of N_2 and CO_2, *Carbon,* **2005**, 91, 330–337.

[8] Crank, J., The mathematics of diffusion. 2nd Edition, *Oxford University Press*., **1979**.

[9] Ruthven, D.M., Lee, L. K., Kinetics of nonisothermal sorption: Systems with bed diffusion control, *AIChE J*. **1981**, 27, 654–663.

[10] Pimentel, B.R., Control of Diffusive Time Scales in Zeolitic Immidazolate Frameowrks for the Kinetic Separation of Light Hydrocarbons Control of Diffusive Time Scale in Zeolitic Imidasolate Frameworks for the Kinetic, *Georgia Institute of Technology*, **2018**.

[11] Ma, Y., Jue, M.L., Zhang, F., Mathias, R., Jang, H.Y., Lively, R.P., Creation of Well-Defined “Mid-Sized” Micropores in Carbon Molecular Sieve Membranes, *Angew. Chem. Int. Ed*. **2019**, 131, 13393–13399.
